# Modularized viromimetic polymer nanoparticle vaccines (VPNVaxs) to elicit durable and effective humoral immune responses

**DOI:** 10.1093/nsr/nwad310

**Published:** 2023-12-07

**Authors:** Zichao Huang, Xinyu Zhuang, Liping Liu, Jiayu Zhao, Sheng Ma, Xinghui Si, Zhenyi Zhu, Fan Wu, Ningyi Jin, Mingyao Tian, Wantong Song, Xuesi Chen

**Affiliations:** Key Laboratory of Polymer Ecomaterials, Changchun Institute of Applied Chemistry, Chinese Academy of Sciences, Changchun 130022, China; School of Applied Chemistry and Engineering, University of Science and Technology of China, Hefei 230026, China; Changchun Veterinary Research Institute, Chinese Academy of Agricultural Sciences, Changchun 130122, China; Key Laboratory of Polymer Ecomaterials, Changchun Institute of Applied Chemistry, Chinese Academy of Sciences, Changchun 130022, China; School of Applied Chemistry and Engineering, University of Science and Technology of China, Hefei 230026, China; Key Laboratory of Polymer Ecomaterials, Changchun Institute of Applied Chemistry, Chinese Academy of Sciences, Changchun 130022, China; School of Applied Chemistry and Engineering, University of Science and Technology of China, Hefei 230026, China; Key Laboratory of Polymer Ecomaterials, Changchun Institute of Applied Chemistry, Chinese Academy of Sciences, Changchun 130022, China; Jilin Biomedical Polymers Engineering Laboratory, Changchun 130022, China; Key Laboratory of Polymer Ecomaterials, Changchun Institute of Applied Chemistry, Chinese Academy of Sciences, Changchun 130022, China; Jilin Biomedical Polymers Engineering Laboratory, Changchun 130022, China; Key Laboratory of Polymer Ecomaterials, Changchun Institute of Applied Chemistry, Chinese Academy of Sciences, Changchun 130022, China; School of Applied Chemistry and Engineering, University of Science and Technology of China, Hefei 230026, China; State Key Laboratory of Polymer Physics and Chemistry, Changchun Institute of Applied Chemistry, Chinese Academy of Sciences, Changchun 130022, China; Changchun Veterinary Research Institute, Chinese Academy of Agricultural Sciences, Changchun 130122, China; Changchun Veterinary Research Institute, Chinese Academy of Agricultural Sciences, Changchun 130122, China; Key Laboratory of Polymer Ecomaterials, Changchun Institute of Applied Chemistry, Chinese Academy of Sciences, Changchun 130022, China; School of Applied Chemistry and Engineering, University of Science and Technology of China, Hefei 230026, China; Jilin Biomedical Polymers Engineering Laboratory, Changchun 130022, China; Key Laboratory of Polymer Ecomaterials, Changchun Institute of Applied Chemistry, Chinese Academy of Sciences, Changchun 130022, China; School of Applied Chemistry and Engineering, University of Science and Technology of China, Hefei 230026, China; Jilin Biomedical Polymers Engineering Laboratory, Changchun 130022, China

**Keywords:** vaccine, polymer, nanomaterials, SARS-CoV-2

## Abstract

Virus-like particle (VLP) vaccines had shown great potential during the COVID-19 pandemic, and was thought to be the next generation of antiviral vaccine technology due to viromimetic structures. However, the time-consuming and complicated processes in establishing a current recombinant-protein–based VLP vaccine has limited its quick launch to the out-bursting pandemic. To simplify and optimize VLP vaccine design, we herein report a kind of viromimetic polymer nanoparticle vaccine (VPNVax), with subunit receptor-binding domain (RBD) proteins conjugated to the surface of polyethylene glycol-*b*-polylactic acid (PEG-*b*-PLA) nanoparticles for vaccination against SARS-CoV-2. The preparation of VPNVax based on synthetic polymer particle and chemical post-conjugation makes it possible to rapidly replace the antigens and construct matched vaccines at the emergence of different viruses. Using this modular preparation system, we identified that VPNVax with surface protein coverage of 20%–25% had the best immunostimulatory activity, which could keep high levels of specific antibody titers over 5 months and induce virus neutralizing activity when combined with an aluminum adjuvant. Moreover, the polymer nano-vectors could be armed with more immune-adjuvant functions by loading immunostimulant agents or chemical chirality design. This VPNVax platform provides a novel kind of rapidly producing and efficient vaccine against different variants of SARS-CoV-2 as well as other viral pandemics.

## Teaser

A polymeric nanoparticle-based two-step strategy was reported to rapidly construct viromimetic vaccines against SARS-CoV-2 or other pandemics.

## INTRODUCTION

The concept of virus-like particle (VLP) vaccines can be traced back to the hepatitis B virus (HBV) vaccine first licensed in 1981 [1], which was based on hepatitis B virus surface antigen (HBsAg) particles secreted by the infecting virus, smaller than the intact virion without much consideration of structural effects [2]. Human papillomavirus (HPV) vaccine, first approved in 2006 [3], is regarded as the most typical and successful VLP vaccine, which consists of icosahedral particles composed of the L1 structural proteins of HPV reassembled *in vitro* [4]. The important feature of the HPV vaccine is that it retains and leverages the assembly capacity of viral structural protein to present the antigen conformational epitopes of the original virus, working as a pathogen-associated structural pattern (PASP) for effective immune system recognition [5], while lacking viral genetic material to produce a vaccine with higher safety and greater acceptability. Afterwards, since not all viral antigenic proteins assemble well *in vitro*, genetically fusing or chemically conjugating target antigen epitopes to structural proteins of different origins to construct chimeric VLPs [2] became the mainstream strategy. However, the assembly process of chimeric VLPs is highly unpredictable, and the development period is generally hampered by problems such as heterogeneous and unstable assembly, suboptimal exposure of introduced antigens and low yields [6,7]. The modular molecular assembly approach is a more robust and flexible strategy for chimeric VLPs [2,8], in which the VLP cores and target antigens are prepared separately and antigens are linked to the surface of the pre-assembled cores either covalently or noncovalently. This post-conjugating method can bypass the unpredictable phase of fusion protein assembly, making the design and preparation of VLP vaccines more controllable.

The COVID-19 pandemic, caused by severe acute respiratory syndrome coronavirus 2 (SARS-CoV-2), was a global catastrophe taking an unprecedented toll on every front [9–11]. While the traditional inactivated vaccines and the rapidly developed mRNA vaccines played a significant role in the early phase, VLP vaccines with higher safety and extended immune stimulation period are thought to be promising for the next generation of vaccines [12]. One of the reasons for the strong infectivity and high pathogenicity of SARS-CoV-2 is that the typical spike structures (S proteins) of coronavirus work as natural barriers against immunosurveillance by extending the neutralizing epitopes at the top and thus reducing their density on the outer surface of the virion [13]. The surface epitopes of SARS-CoV-2 spaced by more than 25 nm rather than the 5–10 nm needed for optimal cross-linking of B cell receptors (BCRs) [14] are inefficient for humoral immune activation. Therefore, reassembling the neutralizing epitopes of SARS-CoV-2 into a VLP vaccine with conventional virus structure is a kind of countermeasure with obvious advantages over other vaccine designs. NVX-CoV2373 (Novavax) was a kind of commercial VLP vaccine [15] that gained widespread attention in the late stages of the COVID-19 pandemic and was put into emergency use in 2021. Assembling as full-length S protein trimers arranged around a polysorbate 80 (PS80) core [16], NVX-CoV2373 is superior to inactivated vaccines because of higher protein activity and more accurate epitope stimulation, and can reach similar protecting efficacy of ∼90% against different variants of SARS-CoV-2 with mRNA vaccines while being safer and more acceptable as a subunit vaccine [17,18]. However, according to WHO data released on March 30, 2023, only 4% of candidate vaccines for SARS-CoV-2 in clinical phase trials are VLP vaccines, while similar types of protein subunit vaccines account for the largest proportion of 32% [19]. The huge gap between protein subunit vaccines and VLP vaccines may be due to the fact that currently most VLP systems are still based on a time-consuming one-step chimeric VLP strategy [20], while the newly developed modular molecular assembly approaches, like VLPs based on Spy-Tag technology [21], require further genetic design and modification of subunit proteins, and are unable to achieve the direct transition from subunit proteins to VLPs thus limiting their ability to be quickly launched in future viral pandemic outbursts.

**Scheme 1.**
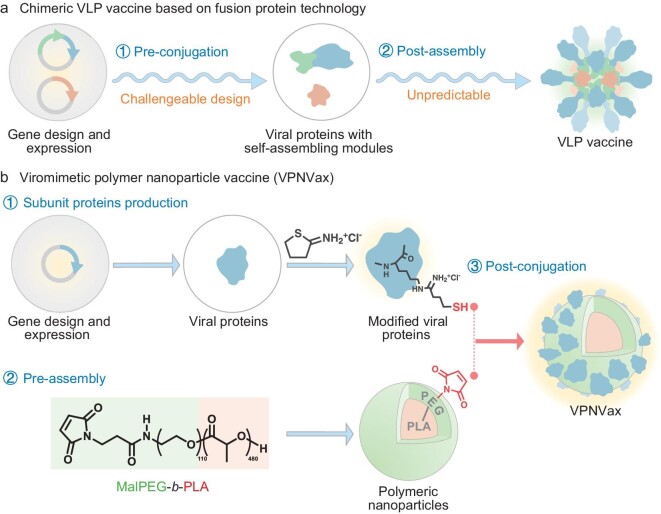
Schematic illustration of the preparation process of chimeric VLP vaccine based on fusion protein technology and VPNVax. (a) Conventional chimeric VLP vaccine first requires the production of fusion viral proteins conjugated with self-assembling modules by challengeable gene design and expression, and then carrying out unpredictable self-assembly of the proteins to get the vaccine particles. (b) VPNVax requires fewer structural designs of the viral proteins and could directly apply the production line of subunit proteins. Viral proteins are modified by Traut's Reagent with part of their amino groups from lysine sites reacted into sulfhydryl groups, followed by post-conjugation onto the surface of the pre-assembly MalPEG-*b*-PLA nanoparticles through click chemical reactions with maleimide groups. This chemical post-conjugation method is more controllable and efficient.

In the present study, we sought to directly reconstruct the subunit proteins of SARS-CoV-2 into VLP vaccine based on synthetic-material particles and click chemical conjugation (Scheme 1). The FDA approved polymer polyethylene glycol-*b*-polylactic acid (PEG-*b*-PLA) was chosen as the material for nanoparticle vectors, with subunit receptor-binding domain (RBD) proteins of SARS-CoV-2 serving as important targets for developing effective vaccines [22], are conjugated onto the surface of pre-assembled PEG-*b*-PLA nanoparticles through the reaction of maleimide with sulfhydryl groups, which generate viromimetic polymer nanoparticle vaccine (VPNVax). The VPNVax platform follows the idea of a two-step modular assembly, making it possible to rapidly replace the antigens and construct matched vaccines at the emergence of different virus variants. Compared to recombinant-protein–based VLPs, polymer nano-vectors have lower heterogenous immunogenicity and can be rapidly mass-produced, they are more flexible, and are able to present sufficient conjugating reaction sites on the surface through simple and controllable chemical design. Moreover, in this strategy, subunit RBD proteins can be stably and efficiently conjugated to the particle surface through rapid chemical modification without additional genetic modification.

VPNVax was proved to successfully mimic the structure of a conventional virus with similar size to SARS-CoV-2 (about 100 nm). VPNVaxs with a series of surface antigen valences could be prepared by controlling the degree of reaction, and we sought to use a kind of mathematical lattice model to fit them to estimate their structural parameters for the study of the structure-activity relationship. Results showed that VPNVax with surface protein coverage of 20%–25% had the best draining lymph node (dLN) trafficking efficiency and specific-antibody stimulating activity. VPNVax with optimized surface valence (oVPNVax) could retain in dLNs for more than 2 weeks and efficiently activate germinal centers (GCs). Specific antibodies elicited by oVPNVax showed advantages of higher titer levels (at the same dose and adjuvant conditions, the endpoint titers of oVPNVaxs were 30–100 times higher than protein subunit vaccines), longer duration (maintaining high titers for more than 5 months) and better antibody quality (increased proportion of IgG2c subtype), and after being formulated with aluminum adjuvant, oVPNVax could induce stronger and more durable virus neutralizing activity of the immunized serum (day 80, mean of NT50 = 200) than protein subunit vaccines (day 40, mean of NT50 = 52). Furthermore, the polymer vectors could be exploited with their unique advantages as synthetic materials. oVPNVax based on hydrophobic assembled micelles could be loaded with hydrophobic immune agonist R848 or IMDQ, which further enhanced specific antibody response and induced a stronger cellular immune response. The chemical chirality of the polymer could be optimized also, and results showed that oVPNVax based on D-type PLA had advantages in activating more IgG2c. Therefore, we anticipate this VPNVax platform could provide a new solution for SARS-CoV-2 as well as other potential new global pandemics.

## RESULTS AND DISCUSSION

### Preparation and characterization of VPNVaxs with different surface valences

The construction idea of VPNVax is first ‘pre-assembly’ based on biodegradable polymer materials, and then ‘post-conjugation’ based on the click reaction between the sulfhydryl group of antigen proteins and maleimide group of nanoparticles (Scheme 1b). Biocompatible PLA was chosen to construct the cores of VPNVax. To introduce the maleimide group, Mal-PEG_5k_-OH was used to initiate the ring opening polymerization of lactide (racemic, D : L = 1 : 1) for synthesis of MalPEG-*b*-PLA polymer (Supplementary Fig. S1a). ^1^H NMR characterizations revealed that the molecular weight of PLA segments was about 32 kDa, and the peak at about 6.7 ppm represented the preservation of maleimide groups (Supplementary Fig. S1b). This amphiphilic polymer can be assembled by nanoprecipitation to prepare nano-micelles with stable properties. Results from Zetasizer Nano ZS showed that the Z-average diameter of PEG_5k_-PLA_30k_ nanoparticles was 103.2 nm with PDI of 0.167 and zeta potential of −23.3 ± 13.3 mV, while its intensity-distribution size was 124.9 ± 43.7 nm and number-distribution size was 73.0 ± 23.1 nm (Supplementary Fig. S2a and S2b).

**Figure 1.**
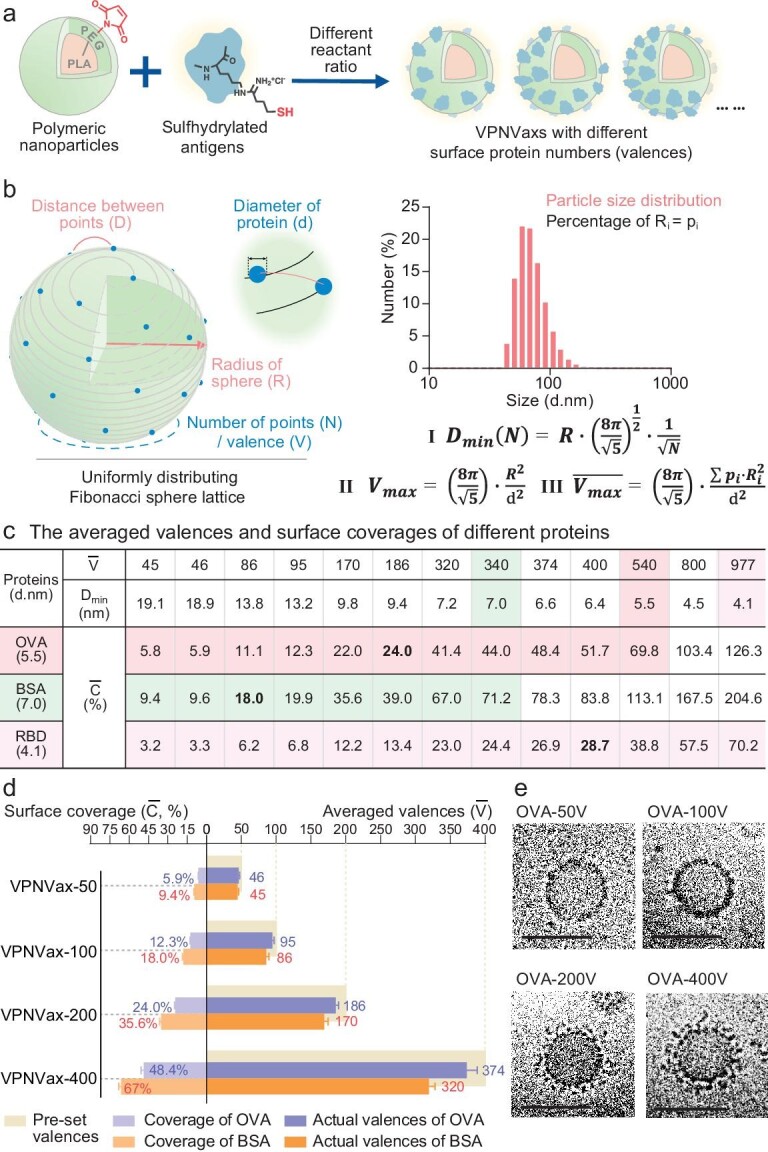
Preparation and characterization of VPNVax. (a) Preparation of VPNVaxs with different surface valences. Antigen proteins were modified by 2-IT for activated sulfhydryl. (b) Schematic illustration of the evenly distributing Fibonacci sphere lattice. Considering the three-dimensional structure of the sphere, the minimum spacing *D*_min_ among evenly distributed points on the sphere can be calculated by Formula-I. When *D*_min_ is equal to the double radius (diameter) of the point (protein), the number (valence) would reach saturation and can calculated by Formula-II; Right panel: application of the lattice algorithm in nanoparticles with size distribution. The number-distribution size histogram of the assembled nanoparticles is shown and its specific data are shown in Supplementary Table S1. The average saturated valence $\overline {{V}_{{\mathrm{max}}}} $ can be calculated by Formula-III. (c) The averaged valences ($\bar{V}$) and corresponding surface coverages ($\bar{C}$) of different proteins with different diameters in Fibonacci sphere lattice. The averaged surface coverage was calculated by Formula-IV. (d) The actual averaged valences and the corresponding averaged surface coverages of VPNVax-OVA/BSA in different pre-set valences. All data are presented as the mean ± SD (*n* = 3). (e) The cryo-EM photographs of VPNVaxs-OVA with pre-set valences of 50, 100, 200 and 400 (scale bar = 50 nm).

Ovalbumin (OVA, ∼44 kDa) and Bovine serum albumin (BSA, ∼66 kDa), two typical heterologous antigenic proteins, were chosen as the model antigen proteins for the following study of VPNVax preparation and function. Antigen proteins were modified by Traut's reagent (2-iminothiolane, 2-IT) to obtain reactive sulfhydryl groups for the conjugation to maleimide group, and the relationship of the excess ratio of 2-IT and conjugating efficiency were determined through a BCA assay kit after purification. As shown in Supplementary Fig. S3, the ratio of 2-IT to protein was optimized at 5 : 1 to avoid over-modifying proteins while still realizing efficient conjugation (>85%). The morphology of purified antigen-nanoparticle conjugates was characterized by cryo-EM (Supplementary Fig. S4) and proved to successfully mimic virus structure with antigen proteins distributing on the particle surface. The ratios of antigen proteins and polymer vectors were the next noteworthy parameters (Fig. 1a). Because the reaction sites on a polymer vector surface are uncountable, and under high-efficiency reaction conditions, the reactant ratio would directly affect the average number of proteins on each particle, which number is referred to as ‘valence’ in this paper, and may ultimately affect the immunization effect of the vaccine. At first, we applied the commonly used two-dimensional grid method [13,23] to calculate the saturated number of proteins that can be conjugated to a unit of nanoparticles with average size (Supplementary Fig. S5a). But we found that the actual conjugating number of proteins could be much higher than the saturated value (Supplementary Fig. S5b), which was attributed to the possible oxidative cross-linking of sulfhydrylated proteins on the particle surface or physical adsorption. The multilayer stacking of proteins on the particle surface leads to an inefficient utilization rate and is not conductive to the study of the structure-activity relationship about surface valence. In view of the fact that the two-dimensional grid method does not consider steric hindrance and the rule of three-dimensional distribution, which could result in overhigh saturated values, we sought to adopt a more accurate model to fit this spherical surface conjugation pattern.

For spherical particle vectors with an average diameter of ∼100 nm, protein particles with diameter smaller than 10 nm conjugated on its surface can be equivalent to mass points distributed on a spherical surface lattice, a situation which can be fitted well by Fibonacci sphere lattice, a model to describe the even and random distribution of points on the spherical surface [24–26] (Fig. 1b). Referring to the formulas of Fibonacci sphere lattice algorithm (Fig. 1b–I, II and III) [24], when the sphere radius *R* and the number *N* of distributed points are confirmed, the minimum spacing *D*_min_ among distributed points could be calculated by Formula-I. In view of steric hindrance and the hydromechanical size (*d*) of proteins, when *D*_min_ approximates the hydromechanical diameter of the protein, the corresponding *N* is equal to the saturated valence (*V*_max_) of the proteins (Formula-II). Moreover, considering the particle size distribution of the actual vector, the average saturated valence ($\overline {{V}_{{\mathrm{max}}}} $) (Formula-III) is adopted eventually and is used to limit the maximum reactant ratios when preparing VPNVaxs of different antigenic proteins. It shows that the average saturated valences estimated from the two-dimensional grid method are almost 40% higher than that from the Fibonacci sphere lattice algorithm (Supplementary Table S2), confirming our former hypothesis and indicating the Fibonacci sphere lattice may be a better fitting model.

Based on the analysis above and different sizes of proteins, the averaged saturated valence of OVA (*d* ∼5.5 nm) [27] and BSA (*d* ∼6.9 nm) [28] can be calculated as 540 and 340, respectively, and structural parameters of VPNVaxs with different valences including antigen spacing (*D*_min_) and surface coverage ($\bar{C}$) could be calculated from the algorithm formulas (Fig. 1c, Formula-IV:${\mathrm{\ }}\bar{C} = \frac{{{S}_{proteins}}}{{{S}_{nanoparticles}}} = \frac{{\bar{V} \cdot \pi {r}^2}}{{4\pi \cdot \sum {p}_i \cdot R_i^2}} \times 100{\mathrm{\% }}$). To be comparable, VPNVaxs of OVA (VPNVax-OVA) and BSA (VPNVax-BSA) were prepared with the same input valences of 50, 100, 200 and 400, and their actual valences and surface coverages were determined and calculated (Fig. 1d). When the pre-set valences increased from 50 to 400, the actual averaged valences of OVA and BSA increased from 46 to 374 and 45 to 320, and the averaged surface coverages increased from 6.0% to 48.7% and 9.3% to 66.2%, respectively. The surface coverages were related to the sizes of the antigenic proteins, so the coverages of OVA with smaller size would be lower than that of BSA under similar valences. Furthermore, the morphology of VPNVax-OVA was characterized by cryo-EM (Fig. 1e), verifying the successful construction of the viromimetic structure of VPNVaxs as antigenic proteins displaying on the particle surface, and the valence control resulted in gradual changes of the density and thickness of antigenic proteins on the particle surface. There was no significant change in the sizes of VPNVaxs-OVA with different valences, but their surface zeta potential increased with the valences (from −23.7 mV to −14.4 mV) (Supplementary Fig. S6).

### Immune stimulation effect of VPNVaxs with different surface valences

**Figure 2.**
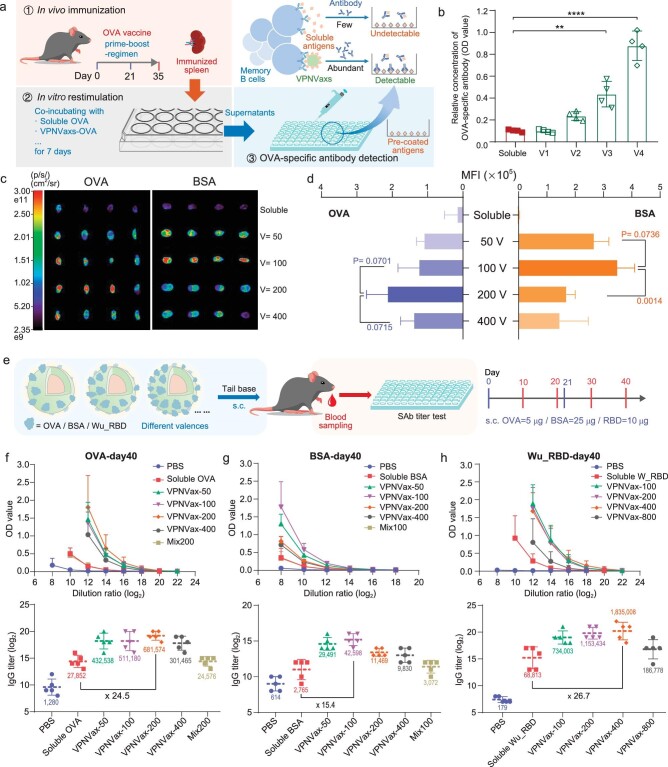
Immune stimulation effect of VPNVaxs with different valences. (a) Schematic illustration of the *in vitro* B cell restimulation by OVA antigen in different formulations. First step: C57BL/6 mouse was immunized subcutaneously in a prime-boost-regimen with OVA vaccine, and its splenocytes containing OVA-specific memory B cells was harvested on day 35. Second step: immunized splenocytes were divided and co-incubated with OVA antigens in different formulations, respectively, for 7 days, when memory B cells would recognize antigens and trigger subsequent antibody response. Third step: OVA-specific antibodies in supernatants were detected through ELISA. (b) Results of the relative concentrations of OVA-specific antibody in supernatants from different groups. The valences of VPNVaxs-OVA were set as 50 (V1), 100 (V2), 200 (V3) and 400 (V4). The relative concentrations were expressed by OD values obtained from ELISA. Data were performed as mean ± SD (*n* = 4) and were analyzed by student's *t*-test. **P < 0.01; ****P < 0.0001. (c) 24h-dLNs-trafficking results of VPNVaxs with different surface valences. *Ex vivo* fluorescence imaging of draining lymph nodes (dLNs) at 24 h post s.c. administered of VPNVax-OVA/Cy5 or VPNVax-BSA/Cy5 with pre-set averaged valences of 50, 100, 200 and 400 at tail base. Scale bar ranged from 2.35e9 to 3.00e11 p/s/cm^2^/sr. (d) Quantification of mean fluorescence intensity of (c). Data were performed as mean ± SD (*n* = 4) and were analyzed by student's *t*-test. (e) Vaccination regimen of VPNVaxs with different antigens and different surface valences. C57BL/6 mice were immunized subcutaneously on day 0 and day 21 at tail base, and their sera were collected on day 10/20/30/40 for antigen-specific antibody (SAb) titer tests. (f) OVA, (g) BSA and (h) Wu_RBD specific IgG antibody endpoint titers of different treatment groups on day 40; Top: the changing curves of absorption intensity (OD value, optical density) vs dilution ratio (log2) of sera in specific antibody titer assay. Bottom: specific IgG antibody endpoint titers on day 40. Endpoint titers derived from antigen-specific antibody titer assays are plotted, with the y-axis representing the log base 2 of titers and average titer values labeled below the plots. All data are presented as the mean ± SD, *n* = 5.

As a type of VLP vaccine, VPNVaxs with surface antigen presentation have the potential to crosslink B cell receptors and activate B cells efficiently to produce antigen-specific antibodies [29]. In order to directly verify this PASP and explore the valence effect of VPNVaxs, we first designed an experiment to stimulate B cells *in vitro*. As shown in Fig. 2a, splenocytes from mice immunized with the same OVA vaccine were co-incubated with soluble OVA or VPNVaxs-OVA with different valences in the presence of IL-2 for 7 days [30], where the OVA-specific memory B cells would recognize antigens and be stimulated to differentiate between them, resulting in the production of OVA-specific antibodies. The relative concentrations of OVA-specific antibodies in the supernatants were detected through the ELISA method. Results showed that VPNVaxs were indeed able to activate B cells better than the soluble antigen, and this activation effect was closely related to their surface valences: the antigen-specific antibody response was enhanced with the increase of the valences (Fig. 2b). A similar pattern was found for physical mixing of nanovaccines with different valences (Supplementary Fig. S7), suggesting the common advantage of nanovaccines displaying surface antigens. This valence advantage could be attributed to the theory that when the spacing of antigens was small enough (optimized as 5–10 nm), BCRs could be appropriately cross-linked for the efficient activation of B cells [14], while as the valence of VPNVaxs increases, the spacing of antigen epitopes will meet the demand (Fig. 1c), after which the smaller spacing may provide a more reaction probability. However, high valences also lead to the issue of particle stability. As shown in Supplementary Fig. S8, though particle sizes of VPNVaxs-OVA with different valences could keep stable at 4°C, the stability of high-valence samples became significantly worse at 37°C. Sizes of VPNVax-OVA with a valence of 400 (V4) became too polydisperse for distribution analysis after incubating at 37°C for 24 hours. This difference in stability also affects the *in vivo* lymph node trafficking efficiencies of VPNVaxs.

Since lymph nodes are organized collections of lymphoid tissue designed to identify and fight infection as filters, trafficking to draining lymph nodes (dLNs) is a critical step for vaccines after subcutaneous injection [31,32]. Researchers have shown that particles with size between 20 and 200 nm have the advantage for trafficking to lymph nodes [33,34], therefore we were further interested in the influence of surface valences on the trafficking efficiency of VPNVaxs. OVA and BSA proteins labeled with Cy5 fluorescent molecules were conjugated onto nanoparticles to prepare VPNVaxs-OVA/Cy5 and VPNVaxs-BSA/Cy5 with different valences, while no significant aggregation-caused quenching (ACQ) effect was detected even at high valences (Supplementary Fig. S9). Results showed that VPNVaxs had significant particle dominance over soluble antigens in dLN 24 h-trafficking experiments (Fig. 2c and d). Interestingly, the trafficking efficiency of VPNVax-OVA/BSA firstly enhanced with the increase of the surface valences and then diminished after reaching a certain peak. Although VPNVaxs with higher valences have fewer particle numbers (Supplementary Table S3) (since protein dose was the fixed variate in the experiment), particle numbers had been ruled out to be the key factor in affecting the trafficking efficiency by addition of blank nanoparticles (Supplementary Fig. S10a and S10b). That means the properties of VPNVaxs, rather than the number of particles, played a vital role in trafficking to lymph nodes. Although high valence can increase the loading efficiency of individual VPNVax particles, which is beneficial for trafficking, the accompanying instability would cause a more negative impact, resulting in the overall diminishing of trafficking of VPNVaxs with high valences. The valences with best trafficking effect of VPNVax-OVA and VPNVax-BSA are 200 and 100, with their corresponding surface coverages of 24.0% and 18.0%, respectively (Fig. 1d), which reveals a possible relationship between the best trafficking effect and an appropriate surface coverage (∼20% to 25%).

Based on this result, we further evaluated the *in vivo* effectiveness of VPNVaxs by testing the antigen-specific antibody titers at serial time points (Fig. 2e). Moreover, for comparison with VLP vaccines based on physical adsorption to form protein crown nanoparticles, we set up a physical mixing group of directly blending antigenic proteins with nanocores. Considering the physical mixing of groups with different valences all had similar lymph node draining performance as the free protein group (Supplementary Fig. S11), a representative physical mixing group with fixed valence was set as control. As shown in Fig. 2f–h and Supplementary Figs S12 and S13, the antibody titer increased as time went by from day 10 to day 40, and VPNVax-200OVA and VPNVax-100BSA, respectively, showed the strongest antibody response on day 40, which was consistent with the optimal valence (20% coverage) from the LN trafficking experiment. Importantly, the physical mixing groups only showed similar antibody titers to soluble OVA/BSA proteins, indicating chemical conjugation is necessary to improve the *in vivo* stimulating activity of antigens.

Then we used RBD domain proteins (∼25 kDa, *d* ∼4 nm) from SARS-CoV-2 of the known original Wuhan strain as the antigens for the construction of VPNVax-RBD. The molar ratio (relative to protein) of Traut's Reagent was set as 5 : 1 to avoid impairing the binding activity of RBD to human angiotensin-converting enzyme 2 (hACE2) (Supplementary Fig. S14). Based on the Fibonacci sphere lattice model and the size of RBD proteins [35,36], the averaged saturated valence of RBD can be calculated as 977 (Fig. 1c), and therefore we prepared VPNVax-Wu_RBD with averaged valences of 100, 200, 400 and 800. VPNVax-100/200/400/800 Wu_RBD with different valences were subcutaneously injected into the tail bases of mice on day 0 and performed prime-boost on day 21 (Fig. 2e), blood samples were then collected from the supraorbital vein on days 10/20/30/40. As shown in Supplementary Fig. S15b, compared to soluble RBD, VPNVax-RBD could effectively elicit RBD-specific antibody responses on day 20 after a single injection, with 10–40 folds increase in the antibody titers from VPNVax100-800. A boost injection on day 21 further increased the antibody titers of both soluble RBD and VPNVax-Wu_RBD (Fig. 2h). However, the antibody titers in the VPNVax-Wu_RBD groups were still much higher than the soluble RBD group, with the highest VPNVax-400Wu_RBD about 25-folds higher than soluble RBD on day 40. The averaged surface coverage corresponding to this optimized valence of VPNVax-Wu_RBD is 28.8% (Fig. 1c), which is close to the optimal surface coverage (20%–25%) from the results of model antigens.

### VPNVaxs with optimal surface valences induced efficient specific antibody responses and had synergistic effect with aluminum *in vivo*

**Figure 3.**
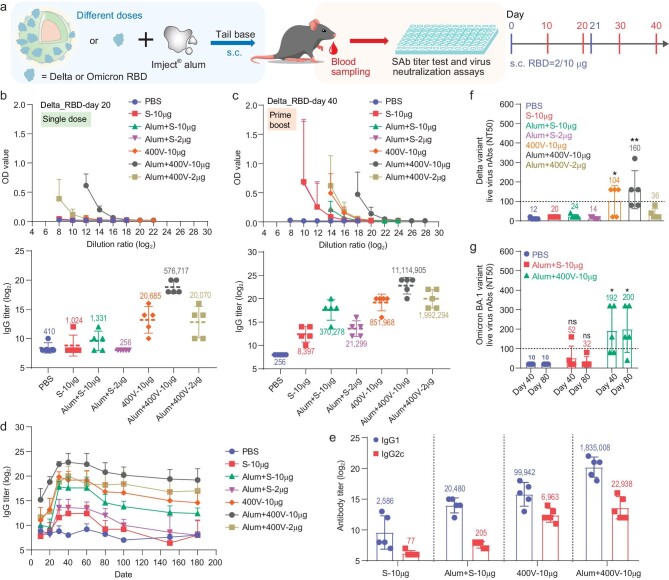
Effect of doses and alum adjuvant on VPNVaxs with optimized valence (oVPNVaxs). (a) Vaccination regimen of oVPNVaxs-RBD in different doses (low dose as 2 μg, normal dose as 10 μg) and formulated with or without alum adjuvant. C57BL/6 mice were immunized on day 0 and day 21 subcutaneously at tail base, and their sera were collected on day 10/20/30/40 for antigen-specific antibody titer tests and authentic SARS-CoV-2 neutralization assays. (b and c) Delta_RBD specific IgG antibody endpoint titers of different treatment groups on (b) day 20 (after single dose on day 0) and (c) day 40 (after prime boost on day 21). S represent soluble proteins, and 400 V represent VPNVax-RBD with optimized valence of 400. Top: the changing curves of absorption intensity (OD value, optical density) vs dilution ratio (log2) of serums in specific antibody titer assay. Bottom: specific IgG antibody endpoint titers of different groups. (d) The changing curve of delta_RBD specific IgG antibody titers of different treatment groups over time. (e) Delta_RBD specific IgG1/IgG2c antibody titers of different treatment groups on day 80. (f) The delta-variant SARS-CoV-2 50% neutralization titers (NT50) of immunized sera from different groups on day 40. The NT50 derived from authentic delta-variant SARS-CoV-2 neutralization assays are plotted, with average NT50 values labeled on the plots. Data were performed as mean ± SD (*n* = 5) and analyzed by student's *t*-test between the selected groups and the PBS group. *P < 0.05; **P < 0.01. (g) The Omicron-variant SARS-CoV-2 50% neutralization titers (NT50) of immunized sera from different groups on day 40 and 80. The NT50 derived from authentic omicron-variant SARS-CoV-2 neutralization assays are plotted, with average NT50 values labeled on the plots. Data were performed as mean ± SD (*n* = 5) and analyzed by student's *t*-test between the selected groups and the PBS group. ns: *P* > 0.05; *P < 0.05.

RBD proteins from the delta (B.1.617.2) variant could easily replace Wu_RBD in preparing VPNVax-D_RBD using the post-conjugation method, and an optimized valence of 400 was selected to construct this oVPNVax. We performed another *in vivo* study using aluminum as the adjuvant for both free delta_RBD and oVPNVax-D_RBD at two different dosages (10 μg and 2 μg) (Fig. 3a). As shown in Fig. 3b and Supplementary Fig. S16a, even with aluminum as the adjuvant, soluble D_RBD groups failed to elicit significant antibody responses after a single dose of vaccine, as opposed to the oVPNVax groups. After a boost injection, the antibody responses of soluble D_RBD groups improved significantly, but at the same dose, the averaged titer of Alum + S-10 μg group could not exceed that of 400V-10 μg group (oVPNVax without aluminum) as well (Fig. 3c and Supplementary Fig. S16b and S16f), with the average titers of 400V-10 μg group and Alum + 400V-10 μg group about 2 times and 30 times that of Alum + S-10 μg group, respectively, on day 40. Of note, oVPNVax-D_RBD could achieve high antibody responses at low doses with the help of aluminum adjuvant (Alum + 400V-2 μg, average titers which was about five times that of the Alum + S-10 μg group on day 40), which is inspiring since the insufficient production capacity of subunit proteins maintains a challenge for the scale production of subunit vaccines [37,38]. Moreover, under monitoring RBD-specific antibody levels over a long time scale (Fig. 3d), it showed that no matter dosage or adjuvant, titers of soluble RBD (S) groups met a significant decline after day 60, while titers of oVPNVax (400V) groups could maintain a fairly high level until day 180. This high-effect and long-term performance of VPNVax is vital in order to deal with the recurrent outbreak of SARS-CoV-2 and its variants.

In addition to quantity, the quality of the antibody elicited by vaccines is important as well [39]. It has been reported that the IgG2 subtype has lower risk to cause potential antibody-dependent enhancement (ADE) side-effects than the IgG1 subtype, and is more conductive to inducing the T helper 1 (Th1)–biased cellular response [33,40]. IgG subtypes against D_RBD were detected on day 80 (Fig. 3e) and it showed that soluble RBD could barely elicit IgG2c, while oVPNVax could elicit a higher percentage of IgG2c. Notably, the ratio of IgG2c titer (log_2_) to IgG1 titer (log_2_) of the alum-adjuvant oVPNVax group (Alum + 400V-10 μg, IgG2c/IgG1 = 0.67) is lower than that of the pure oVPNVax group (400V-10 μg, IgG2c/IgG1 = 0.79), which may reveal that oVPNVax alone has the advantage to elicit IgG2c but the enhancement from aluminum adjuvant results more in generating IgG1.

Next, the delta_RBD immunized sera from different groups were used for the authentic delta-variant SARS-CoV-2 neutralization assays (Fig. 3f). Results showed that the overall 50% neutralization titers (NT50) of soluble RBD groups (S-10 μg, Alum + S-10 μg and Alum + S-2 μg) were nearly undetectable (1 : 10 to 1 : 40), while NT50 of oVPNVax groups (400V-10 μg, Alum + 400V-10 μg and Alum + 400V-2 μg) were relatively high (1 : 20 to 1 : 320). NT50 of 400V-10 μg and Alum + 400V-10 μg were about 4- and 6-fold higher than Alum + S-10 μg, indicating the ability of oVPNVax to enhance virus neutralizing activity. Furthermore, RBD proteins from the omicron (BA.1.1) variant, which variant was quickly identified as being significantly more transmissible than delta and replaced delta as the dominant variant in 2021, was reconstructed as oVPNVax with surface valence of 400 and the immunized sera were collected to evaluate the virus neutralization ability. As shown in Fig. 3g, soluble RBD with alum could not elicit enough NT50 (mean = 52). By contrast, oVPNVax with alum could elicit a 4-fold higher NT50 (mean = 200) than the soluble group on day 40, and kept this level until day 80. The rapid shift from delta-vaccine to omicron-vaccine demonstrated the advantage of VPNVax against the emergence of multiple variants.

### Retention and immunostimulation of oVPNVax in draining lymph node

**Figure 4.**
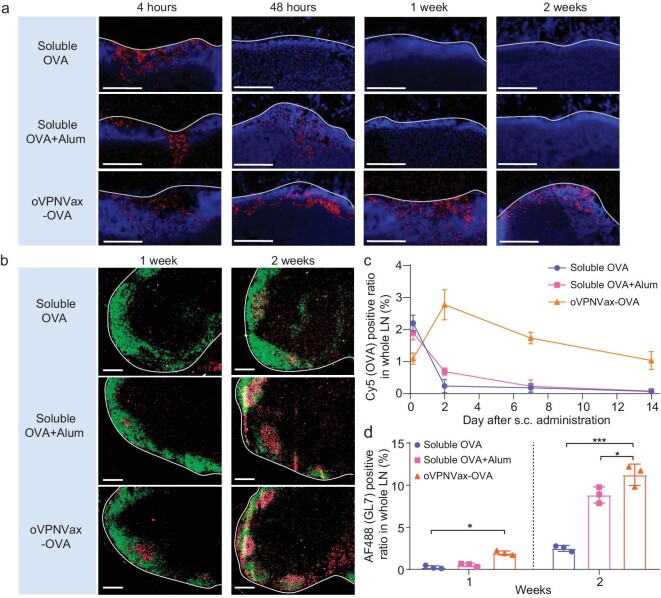
Mechanism for effective and durable effect of oVPNVaxs: retention and immunostimulation of oVPNVax in draining lymph node. (a) CLSM images of OVA/Cy5 distribution in lymph nodes of different treatment groups at different time points (Scale bar = 2 μm). OVA/Cy5 channel is set as red while DAPI channel is set as blue in the images, and drawn white lines indicate the outer contour of lymph nodes. (b) CLSM images of GCs activation in lymph nodes of different treatment groups at different time points (Scale bar = 2 μm). GC B cells (GL7^+^) channel is set as red while B cell follicles (B220^+^) channel is set as green in the images. Drawn white lines indicate the outer contour of lymph nodes. (c) Quantification of OVA/Cy5 positive ratio in whole LN of different treatment groups at different time points. All data are presented as the mean ± SD (*n* = 3). (d) Quantification of GL7 positive ratio in whole LN of different treatment groups at different time points. Data were performed as mean ± SD (*n* = 3) and analyzed by student's *t*-test. *P < 0.05; ***P < 0.001.

The efficient immunostimulating ability of VLP vaccines is usually related to their long-term retention and robust stimulation in lymph nodes [41–43]. After reaching the lymph nodes, the retention time of antigens determines whether they can induce durable and high-affinity humoral immune responses [33]. We immunized mice with Cy5-labeled OVA (soluble OVA, soluble OVA formulated with aluminum adjuvant or oVPNVax-OVA) and performed immunofluorescence analyses of dLNs at serial time points from 4 h to day 14 after injection (Fig. 4a and Supplementary Fig. S17). Both soluble and conjugated OVA were detected in the dLNs at 4 h after injection. Soluble OVA was rapidly cleared within 24 h, while oVPNVax-OVA could be detected until 14 days after injection (Fig. 4c). This extended retention and distribution pattern of nano-vaccines has been proven to facilitate their capture by follicular dendritic cells (fDCs) and to keep long-time sufficient contact with B cells, thus inducing more robust humoral immune responses [44,45], which annotate the long-term specific antibody responses induced by oVPNVaxs (Fig. 3d).

Since germinal center (GC) is the site for somatic hypermutation (SHM) of B cells and diversification and affinity maturation of antibodies [46,47], the rapid and robust generation of GCs is the key hallmark of effective vaccination. Therefore, we further evaluated the GC in the LNs after various vaccine treatments. C57BL/6 mice were immunized with soluble OVA, soluble OVA formulated with aluminum adjuvant or oVPNVax-OVA, and immunofluorescence analyses of B cell zones (labeled by B220/AF594, green) and GCs (labeled by GL7/AF488, red) in dLNs were carried out at serial time points from 4 h to day 14 after injection. As shown in Fig. 4b and d and Supplementary Fig. S18, without the assistance of adjuvants, soluble OVA could not induce obvious GCs before the first week with only modest emerging of GCs on day 14. In contrast, oVPNVax-OVA elicited visible GCs on day 7 and a more robust response on day 14, with GL7 positive ratio nearly 3-folds higher than that of the soluble OVA group and 1.5-folds higher than that of soluble OVA formulated with aluminum adjuvant. This rapid and robust GC activation explained the effective specific antibody responses elicited by oVPNVax after a single dose (Fig. 3b). Moreover, adjuvants are generally necessary for eliciting B cell activation and GC generation [48–50]. However, B cells could also be activated by nanostructures displaying with repeated antigens through crosslinking of the B-cell receptors (which is also called a geometry-associated molecular pattern, GAMP). In view of the bionic design philosophy of VPNVax, the viromimetic surface of VPNVax could stimulate GC generation through a self-adjuvating manner.

### Immune potency of oVPNVax enhanced by optimal design of polymeric vectors

**Figure 5.**
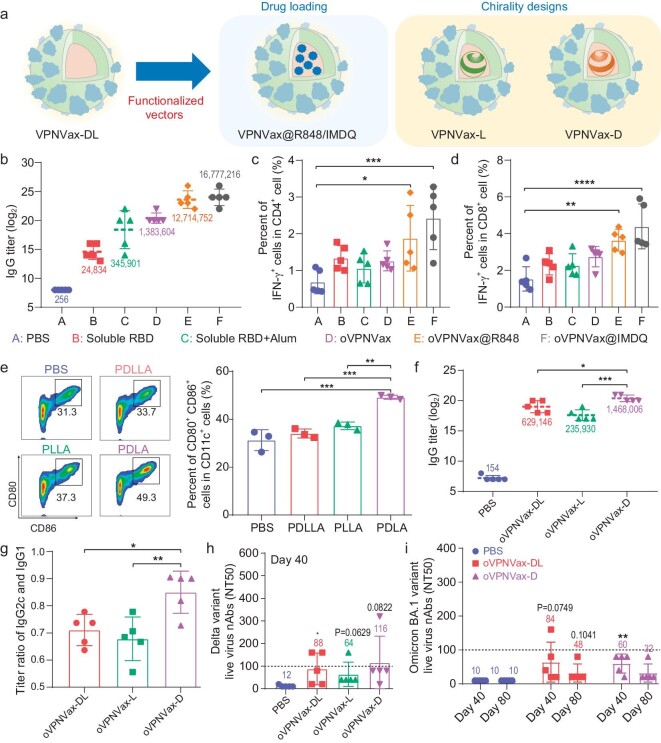
Development of functionalized vectors for oVPNVaxs by drug loading and chirality design. (a) Schematic diagram of functionalized vectors of oVPNVaxs. Drug loading: oVPNVax based on hydrophobic assembled micelles could be loaded with hydrophobic immune drugs like R848 or IMDQ, both of which are TLR7/8 agonist. Chirality designs: three types of PLA can be prepared by adjusting the ratio of the two chiral monomers (L-LA and D-LA), which may influence the self-immunogenicity of PLA. DL: racemic (50%L-LA + 50%D-LA), L: laevogyrate (100%L-LA), D: dextrogyre (100%D-LA). (b) Delta_RBD specific IgG antibody endpoint titer induced by different groups, including oVPNVax loaded with R848 (oVPNVax@R848) or IMDQ (oVPNVax@IMDQ). C57BL/6 mice were immunized on day 0 and day 21 and serum samples were collected on day 20 and day 40 for antibody titers assay. Endpoint titers derived from antigen-specific antibody titer assays are plotted, with the y-axis representing the log base 2 of titers and average titer values labeled below the plots (*n* = 5). (c and d) The percentage of activated (c) CD4^+^ T cells and (d) CD8^+^ T cells (IFNγ^+^) in blood after immunized by different vaccines. C57BL/6 mice were immunized on day 0 and blood samples were collected on day 7 for flow cytometry analysis (*n* = 5). (e) Left panel: representative flow cytometry scatter plots, gated on CD11c^+^ in BMDC, are shown with the percentages of CD86^+^CD80^+^ populations representing activated ratios of BMDC. Materials labeled on each pane were co-incubated with BMDC for 6 h, followed by flow cytometry analysis. Right panel: statistic of activated populations (CD86^+^CD80^+^) in BMDC treated with different materials (*n* = 3). (f) Delta_RBD specific IgG antibody titers of different treatment groups on day 40. C57BL/6 mice were immunized with different chirality types of delta_RBD vaccines on day 0 and day 21. The values labeled under or above each set of data represent the average value of that set of data (*n* = 5). (g) Statistic of ratios of delta_RBD specific IgG2c antibody titers to IgG1 antibody titers (IgG2c/IgG1) (*n* = 5). oVPNVax based on PDLA had an advantage in activating more IgG2c. Data above were performed as mean ± SD and analyzed by student's *t*-test. *P < 0.05; **P < 0.01; ***P < 0.001; ****P < 0.0001. (h) The delta-variant SARS-CoV-2 50% neutralization titers (NT50) of immunized sera from different groups on day 40. The NT50 derived from authentic delta-variant SARS-CoV-2 neutralization assays are plotted, with average NT50 values labeled on the plots. Data were performed as mean ± SD (*n* = 5) and analyzed by student's *t*-test between the selected groups and the PBS group. (i) The omicron-variant SARS-CoV-2 50% neutralization titers (NT50) of immunized sera from different groups on days 40 and 80. The NT50 derived from authentic omicron-variant SARS-CoV-2 neutralization assays are plotted, with average NT50 values labeled on the plots. Data were performed as mean ± SD (*n* = 5) and analyzed by student's *t*-test between the selected groups and the PBS group.

Using PLA as the core provides other possibilities to further improve the effectiveness of VPNVax (Fig. 5a). First, the hydrophobic PLA nanocore could be loaded with immune adjuvants to further enhance the immunostimulation effect. Second, as synthetic material, PLA vectors can be endowed with more functions through definite and controllable chemical molecular designs such as chirality regulation.

Toll-like receptor 7/8 agonists had been widely developed as a vaccine adjuvant and was proved to have the advantage to elicit Th1-type immune responses [51], and imidazoquinoline derivatives are one of the most commonly used small molecule TLR7/8 agonist family [52]. R848 (resiquimod) and IMDQ (imidazoquinoline with aminomethyl-benzyl modification [52,53]), both of which are imidazoquinoline derivatives, were chosen to be loaded into the hydrophobic PLA nanocore for the preparation of oVPNVax@R848 and oVPNVax@IMDQ (Fig. 5a and Supplementary Fig. S19), and they were used to immunize mice as in the previous regimen (prime on day 0 and boost on day 21). Results showed that both RBD-specific antibody titers elicited by oVPNVax@R848-RBD and oVPNVax@IMDQ-RBD were about 10-fold higher than oVPNVax-RBD group on day 40 (Fig. 5b and Supplementary Fig. S20). Moreover, oVPNVax@R848 and oVPNVax@IMDQ could induce more IFN-γ producing CD4^+^ T cells, which is the key indicator of Th1-basis responses, and the corresponding frequency of IFN-γ^+^CD8^+^ T cell was increased as well (Fig. 5c and d, and Supplementary Fig. S21). These results demonstrate that developing the drug loading capacity of vectors can further improve the immune effectiveness of VPNVaxs.

Based on the flexible and changeable chemical designs, the synthetic-material vector of VPNVaxs can be leveraged as a function module as well. Considering the fact that most proteins in organic nature are entirely composed of L (levogyration)-type amino acid while nucleic acids and carbohydrates are almost entirely composed of D (dextrorotation)-type sugars [54], chirality of biomaterials is an important parameter affecting their fate *in vivo* because different chirality of materials would lead to their affinity or rejection with different chiral biomolecules, which could result in some special immune responses [55–57]. Therefore, we adjusted the ratio of chiral monomers in PLA and synthesized PLLA and PDLA containing 100% L-LA and D-LA, respectively, (Supplementary Fig. S1c and S1d). The particle vectors assembled with different chiral types of PLA (Supplementary Fig. S22) were first tested for their direct activation effect on BMDC to explore their auto-immunogenicity. As shown in Fig. 5e and Supplementary Fig. S23, the racemic- and L-type PLA could barely activate BMDC while D-type PLA induced the highest activation level (evaluated by double positive ratios of CD80&CD86). Afterwards, the corresponding oVPNVaxs (oVPNVax-DL, L, D) were prepared and immunized mice with the previous regimen, and immunized sera were collected on days 40/80 for the antigen-specific antibody titer tests and authentic SARS-CoV-2 neutralization assays. Results showed that the RBD-specific IgG titer of the oVPNVax-D group is significantly higher than other groups (Fig. 5f), with about 2-folds and 6-folds higher than oVPNVax-DL and oVPNVax-L groups, respectively. More strikingly, the ratio of IgG2c titer (log_2_) to IgG1 titer (log_2_) of the oVPNVax-D group (IgG2c/IgG1 = 0.85) is higher than that of the oVPNVax-DL group (IgG2c/IgG1 = 0.71) and oVPNVax-L group (IgG2c/IgG1 = 0.68) (Fig. 5g), revealing that oVPNVax made of PDLA could elicit a higher percentage of IgG2c. These results demonstrate that the synthetic polymer vectors of VPNVaxs also have the potential to be further designed and developed for their optimized immune stimulation effects. However, the NT50 induced by oVPNVax-D showed no advantage when compared with the oVPNVax-DL group, either for the delta or omicron variants (Fig. 5h and i), and the overall NT50 levels were low, suggesting the necessity of proper immune adjuvant for oVPNVax to induce virus neutralization activity.

## CONCLUSION

Though the emergency phase of COVID-19 is over, the pandemic has not come to an end [58]. Breakthrough infections and even reinfections have become increasingly common recently [59], which is not only related to the high mutation rate of SARS-CoV-2, but also its structural characteristics that evade immune surveillance. Conventional influenza virus particles have a size of 80–120 nm with ∼700 homotrimeric hemagglutinin (HA) on the surface [60], while SARS-CoV-2 has a size of 60–160 nm with around 25 spike proteins (S proteins) resulting in surface neutralizing epitopes spacing of about 25 nm [35,36,61]. Considering that the optimal epitope spacing is 5–10 nm to efficiently cross-link B-cell receptors (BCRs) and enhance antibody binding [13], SARS-CoV-2 is easier to avoid recognition and clearance by the immune system. Therefore, reassembling the neutralizing epitopes of SARS-CoV-2 into a VLP vaccine is a kind of countermeasure with structural advantage over other vaccine designs. However, the deployment of VLP vaccines during the COVID-19 pandemic had been limited by their challenging designs and low manufacturing efficiency [12,62]. By contrast, the similar type of protein subunit (PS) vaccines accounted for the largest proportion (>30%) of all SARS-CoV-2 vaccines entering the clinical trials phase. The main difference between the two types of vaccines is that most of the current development of VLP mainly followed the idea of a one-step method, with the production objects being the final particles rather than the simple subunit proteins. It takes time to achieve the stable regulation of the unpredictable assembly performance, and sometimes requires relatively high-threshold production systems [2,20]. Therefore, we sought to directly reconstruct the subunit proteins into virus-like particles based on synthetic-material particles and click chemical conjugation. Chemical cross-linking is a common method for binding antigens and native VLPs [2]. However, since native VLPs are usually protein particles, their surface reaction sites are uncontrollable, making chemical conjugating methods unable to control the quantity or orientation of the surface antigens [20]. In this study, synthetic-polymer particles which could present abundant reactive groups on the surface through simple chemical designs were used as VLP vectors. Furthermore, we proposed for the first time to apply the Fibonacci sphere lattice model to fit this process, the significance of which is not only that it helped us to successfully regulate the surface valences of VPNVaxs in this work, but also that it can be used as a general method with standard principles for later VLP vaccine designs.

In conclusion, in this work we successfully reconstructed the subunit proteins RBD of SARS-CoV-2 into VLP vaccines (VPNVax) based on synthetic-polymer particles and click chemical conjugation. This modular preparation method makes it possible to rapidly replace the antigens and construct matched vaccines at the emergence of different viruses. We proposed to apply the Fibonacci sphere lattice model to fit this VLP structure and successfully regulated the surface valences of VPNVaxs. VPNVax with surface protein coverage of 20%–25% was optimal and proven to have high-efficiency and sustained *in vivo* activity to activate RBD-specific antibody responses, and could induce serum virus neutralizing activity when formulated with an aluminum adjuvant. Furthermore, VPNVax vectors based on synthetic polymer have the potential to expand their immune functions by loading immune drug or chemical molecular designs, while loading R848 or IMDQ and dextro-chirality design enhancing the Th1-type immune responses of VPNVaxs was proven in this work. We anticipate this VPNVax platform has the potential to be widely developed and applied for COVID-19 as well as other pandemics, while the idea and construction methods of VPNVax have guiding significance for the next generation of VLP vaccines.

## Supplementary Material

nwad310_Supplemental_File
