# Supplementary material for: Modularized viromimetic polymer nanoparticle vaccines (VPNVaxs) to elicit durable and effective humoral immune responses
Source: Natl Sci Rev. 2023 Dec 7;11(3):nwad310. doi: 10.1093/nsr/nwad310 (PMC10833449; doi:10.1093/nsr/nwad310)
Supplement: nwad310_Supplemental_File [file nwad310_supplemental_file.pdf]

Modularized viromimetic polymer nanoparticle vaccines (VPNVaxs) to elicit durable and effective humoral immune responses

Zichao Huang[a,b], Xinyu Zhuang[c], Liping Liu[a,b], Jiayu Zhao[a,b], Sheng Ma[a,d], Xinghui Si[a,d], Zhenyi Zhu[a,b], Fan Wu[e], Ningyi Jin[c], Mingyao Tian[c,*], Wantong Song[a,b,d,*], Xuesi Chen[a,b,d,*]

[a]Key Laboratory of Polymer Ecomaterials, Changchun Institute of Applied Chemistry, Chinese Academy of Sciences, Changchun, 130022, China

[b]School of Applied Chemistry and Engineering, University of Science and Technology of China, Hefei, 230026, China

[c]Changchun Veterinary Research Institute, Chinese Academy of Agricultural Sciences, Changchun, 130122, China

[d]Jilin Biomedical Polymers Engineering Laboratory, Changchun, 130022, China

[e]State Key Laboratory of Polymer Physics and Chemistry, Changchun Institute of Applied Chemistry, Chinese Academy of Sciences, Changchun 130022, China

Materials and Methods

Materials

D-, and L-Lactide monomer was kindly donated by Changchun SinoBiomaterials Co. Ltd. RBD proteins of SARS-CoV-2 of Wuhan strain and Delta variant were kindly donated by Changchun Veterinary Research Institute. Anhydrous methylbenzene was obtained through vacuum distillation. Anhydrous ethyl acetate was purchased from Beijing Energy Chemical Co. Ltd. Tin 2-ethylhexanoate, L-Cysteine hydrochloride monohydrate and 2-Iminothiolane hydrochloride was purchased from Shanghai Aladdin Co. Ltd. Maleimide PEG Hydroxyl (5 kDa) was purchased from Beijing JenKem Technology Co. Ltd. ACE2 Protein, Human, Recombinant (His Tag) (Cat. 10108-H08B), Anti-His tag Antibody (HRP), Mouse Monoclonal (Cat. 105327-MM02T-H), SARS-CoV-2 Spike RBD (RBM) Peptide Pool (Cat. PP002-A) and SARS-CoV-2 (BA.1.1) Spike RBD Protein (His Tag) (Cat. 40592-V08H129) were purchased from Sino Biological Inc. Ovalbumin (OVA, Cat. A5503) was purchased from Sigma-Aldrich. Bovine Serum Albumin V (BSA, Cat. A8020) was purchased from Beijing Solarbio Science & Technology Co. Ltd. Imject Alum adjuvant (Cat. 77161), Goat anti-Mouse IgG (H+L) Secondary Antibody, HRP (Cat. 31430) and BCA Protein Assay Kit (Cat. 23225) were purchased from Thermo Fisher Scientific. Goat Anti-Mouse IgG1 (HRP) (Cat. ab97240) and Goat Anti-Mouse IgG2c heavy chain (HRP) (Cat. ab97255) were purchased from Shanghai Abcam Co. Ltd. 3, 3′, 5 ,5′-Tetramethylbenzidine (TMB, Cat. P0209) and Antifade Mounting Medium with DAPI (Cat. P0131) were purchased from Shanghai Beyotime Biotechnology Co. Ltd. Recombinant Murine GM-CSF (Cat. 315-03), Recombinant Murine IL-4 (Cat. 214-14) and Recombinant Murine IL-2 (Cat. 212-12) were purchased from PeproTech Inc. R848 (CAS: 144875-48-9) and IMDQ (CAS: 1258457-59-8) were purchased from Suzhou Nuobeike Biotechnology Co. Ltd.

Optimal cutting temperature compound (OCT compound) was purchased from Wuhan Servicebio Co. Ltd. The ultrafiltration devices with MWCO of 10 kDa was Amicon Ultra-0.5 Centrifugal Filter Unit (Cat. UFC5010) purchased from Millipore Sigma Co. Ltd. The ultrafiltration devices with MWCO of 300 kDa was Vivaspin® 500

Centrifugal Concentrators (Cat. VS0152) purchased from Sartorius Co. Ltd. Sulfo-Cy5- succinimidyl ester (Cy5-NHS) was purchased from Meilun Biotechnology Co. Ltd and was used to labeled OVA and BSA proteins and purified by 10 kDa ultrafiltration devices. Cell activation cocktail (with Brefeldin A) (Cat. 423303), intracellular staining permeabilization wash buffer (Cat. 421002), FITC anti-mouse CD3 Antibody (Cat. 100204), PE/Cyanine7 anti-mouse CD4 Antibody (Cat. 100422), APC anti-mouse CD8a Antibody (Cat. 100712), PE anti-mouse IFN-γ Antibody (Cat. 505808), Alexa Fluor® 594 anti-mouse/human CD45R/B220 Antibody (Cat. 103254), Alexa Fluor® 488 anti-mouse/human GL7 Antigen (T and B cell Activation Marker) Antibody (Cat. 144612), APC labeled anti-CD80 (Cat. 104714) and PE/Cyanine 7 labeled anti-CD86 (Cat. 105014) were purchased from Biolegend Inc. Other reagents and solvents were provided by Sinopharm Chemical Reagent Co. LTD.

Characterization

^{1}H NMR spectrum were characterized by Bruker AV-300 NMR spectrometer. Diameter and zeta potential of nanoparticles were measured by Zetasizer Nano ZS (Malvern analytical Co. Ltd). The number concentrations (particles/mL) of nanoparticles were measured by NanoSight NS300 (Malvern analytical Co. Ltd). We thank the Department of Nanomedicine Translator Center of China-Japan Union Hospital of Jilin University for their assistance in the detection of particle sizes and number concentrations. JEOL JEM-1011 (Tokyo, Japan) was used to obtain transmission electron microscopy (TEM) images. JEOL JEM-3200FSC (Tokyo, Japan) was used to obtain cryogenic transmission electron microscopy (cryo-TEM) images. The absorbances of samples in 96-well plates were measured by the SPARK® multimode microplate reader. The *ex vivo* fluorescence images of major organs and tumors were picture with Davinch Invivo Hr imaging system (DavinchK, Korea). All the immunofluorescence slides and cellular uptake slides were pictured through a confocal laser scanning microscope (CLSM, Carl Zeiss LSM 700, Germany). Flow cytometry tests were performed by a flow cytometer (BD FACSCelesta).

Cells and animal use

Bone marrow-derived dendritic cells (BMDCs) were extracted from bone marrow of C57BL/6 mice. Briefly, femurs and tibias were removed out from 4 to 6 weeks old C57BL/6 mice and the bone marrow cells were isolated. Then these cells were incubated in RPMI-1640 containing 20 ng/mL GM-CSF and 10 ng/mL IL-4 for 6 days, during which half of the media were replaced with fresh RPMI-1640 twice and cytokines were supplemented. The BMDCs were harvested at day 7 for further usage.

All animal procedures were performed following the guidelines approved by the Animal Welfare and Ethics Committee of Changchun Institute of Applied Chemistry, Chinese Academy of Sciences (2022-0026). Female C57BL/6 mice (6-8 weeks old) were purchased from Beijing Vital River Laboratory Animal Technology Co. Ltd. (Beijing, China).

Synthesis and characterization of maleimide-terminated polyethylene glycol-*block*-poly (racemic-, D-, or L-lactide acid) (MalPEG-*b*-P (DL, D, or L) LA).

To synthesize MalPEG-*b*-P (DL, D, or L) LA, racemic-, D- or L-lactide monomers were first purified by recrystallization. Briefly, mixture of D-lactide and L-lactide (w: w = 1: 1), D-lactide or L-lactide (30 g) were fully dissolved in 30 mL anhydrous ethyl acetate at 90 ℃ water bath, and then maintained at room temperature for cooling crystallization. The recrystallization repeated twice more and the purified monomers were obtained as white solid after complete removal of ethyl acetate through vacuum drying.

Purified racemic-, D- or L-lactide monomers (700 mg, 4.86 mmol), maleimide PEG_{5k} hydroxyl (Mal-PEG_{5k}-OH, 100 mg, 0.02 mmol) were dissolved in 2.4 mL anhydrous methylbenzene in glovebox, and stannous octoate (1.5 mg, diluted in anhydrous methylbenzene as 20 mg/ml) was added into the mixture solution in nitrogen atmosphere, following sealed and stirred at 110 ℃ for 24 hours. After reaction, the mixture was precipitated into 10 folds volume excess cold diethyl ether and then was

re-dissolved in dichloromethane and repeated precipitation twice more. The purified polymers MalPEG-b-P (DL, D, or L) LA were obtained as white with slightly yellow solid after complete removal of diethyl ether through vacuum drying. The structures were determined by ^{1}H NMR using chloroform-d as the solvent.

Preparation and characterization of MalPEG-*b*-PLA nanoparticles.

The MalPEG-*b*-PLA nanoparticles were prepared by nanoprecipitation method. Typically, for MalPEG-*b*-PDLLA NP preparation, 35 mg MalPEG-*b*-PDLLA was dissolved in 1.5 mL acetone, then the solution was dropwise added to 6 mL deionized water stirring with ice bath. The mixture was then directly dialyzed against DI water (MWCO = 35 kDa) for 24 hours, after which the purified nanoparticle solution was collected and preserved at 4 ℃. MalPEG-*b*-PDLLA NP loaded with R848 or IMDQ were prepared in a similar way, except that 10 wt% (relative to polymer) R848 or IMDQ dissolved with DMSO were added to the acetone phase. For MalPEG-*b*-PDLA or MalPEG-*b*-PLLA NP preparation, 35 mg MalPEG-*b*-PDLA or MalPEG-*b*-PLLA was dissolved in 1.8 mL dioxane, then the solution was dropwise added to 5 mL acetone in vortex, after which the organic solution was dropwise added to 12 mL DI water stirring with ice bath. The mixture was then directly dialyzed against DI water (MWCO = 35 kDa) for 24 hours, after which the purified nanoparticle solution was collected and preserved at 4 ℃. The final mass concentration of the nanoparticle solution was determined through partly lyophilization and accurately weighing. Diameter and zeta potential of nanoparticles were measured by Zetasizer Nano ZS (Malvern analytical Co. Ltd), while TEM and cryo-EM were used to characterize the morphology. The particle number concentrations of initial particle solutions and concentrated particle solutions were measured by NanoSight NS300 (Malvern analytical Co. Ltd).

Modification and purification of antigenic proteins.

Before post-conjugation, antigenic proteins need to be modified by Traut's reagent and purified by centrifugal ultrafiltration. The excess molar ratio of Traut's reagent

was adjustable and could be set as 2/ 5/ 10/ 20 folds. Specifically, 180 μg RBD proteins (25 kDa, 7.2 nmol, 3.6 μg/μL) were diluted with 100 μL PB buffer (pH = 8.0-8.5), followed by addition of 2/ 5/ 10/ 20 μg Traut's reagent (2-Iminothiolane hydrochloride, 137.63 Da, 14.4/ 36/ 72/ 144 nmol, 1 μg/μL in PB) and incubation at room temperature for one hour. The modified proteins were then purified through ultrafiltration (MWCO = 10 kDa) using DI water as eluent, and finalized to a concentration of 1.8 μg/μL, which could be precise quantification by BCA protein assay kit (Thermo Fisher Scientific). The modification and purification of other proteins including OVA and BSA were similar to the procedure introduced above.

Modified RBD proteins activity assay

The binding activity of modified RBD to human ACE2 was tested through Enzyme-linked immunosorbent assay. Briefly, RBD proteins modified by different ratio of Traut's Reagent (0:1, 2:1, 5:1, 10:1, 20:1, 50:1, 100:1) were pre-immobilized on ELISA plate at 10 μg/mL (100 μL/well, incubated overnight at 4 ℃). After washed three times with wash buffer containing PBS and 0.05% (v/v) Tween 20, each well was then coated with 200 μL of blocking buffer consisting of PBS and skim milk powder (20 mg/mL) for two hours at room temperature. After washed three times with wash buffer, 100 μL of binding buffer consisting of PBS and human ACE2 proteins with His tag (40 ng/mL; SinoBiological) were added to each well, followed by incubation for one hour at room temperature. Then the plate was washed three times with wash buffer, and 100 μL of a 1:10000 dilution of horseradish peroxidase (HRP)–conjugated mouse anti-His tag antibody (2.1 μg/μL; SinoBiological) made in blocking buffer was added to each well. The plate was incubated with the antibody for one hour at room temperature and then washed five times with wash buffer. Last, 100 μL of tetramethylbenzidene (TMB) (Beyotime) was added to each well for 15 mins at room temperature before stopping the reaction with 100 μL of 2N sulfuric acid. The resulting plate readouts were measured at a wavelength of 450 nm. The averaged absorption value from group of naïve RBD protein without modified by Traut's reagent (0:1) was set as 100% activity standard.

Preparation and characterization of nanoparticle-protein conjugates.

The fresh modified proteins were added into the nanoparticle solution for conjugation, and the conjugating efficiency was measured through BCA protein assay after removal of unconjugated proteins. Briefly, OVA proteins modified by five excess molar ratios of Traut's reagent were purified and quantified by BCA protein assay. For nanoparticles with normalized mass of 2 mg, different input mass (from 1 μg to 115 μg) of modified OVA proteins were added into the nanoparticle solutions respectively, followed by incubation at 4 ℃ overnight. The mixtures were then purified through ultrafiltration (MWCO = 300 kDa) using DI water as eluent, and finalized to a volume of 200 μL. The final conjugated OVA protein concentrations were quantified by BCA protein assay (in some cases, when the protein input mass was too low for accurate measurement, the actual nanoparticle mass was set as 5-8 mg), and the morphology of purified nanoparticle-protein conjugates were characterized by cryo-EM.

Preparation and characterization of VPNVax with different valences.

The preparation of viromimetic polymer nanoparticle vaccine (VPNVax) was similar to that of nanoparticle-protein conjugates, other than the antigenic proteins input amount were controlled by Fibonacci sphere lattice algorithm and the size of nanoparticles and proteins. Specifically, when the hydromechanical diameter of the antigenic proteins (d) and the number-distribution size curve of the assembled nanoparticles were known, the averaged saturated valence ($\overline{V_{max}}$) can be calculated by Formula III, whose practical meaning is that for given number of the nanoparticles (N_n) conforming to this size distribution, the saturated number of the antigenic protein for single-layer surface conjugation can be calculated as $\overline{V_{max}} \cdot N_n$. The relationship between number and mass of nanoparticle can be tested out through NanoSight NS300, and that of protein can be calculated using molecular weight and the Avogadro constant (N_A). Therefore, in the conjugation experiment of Fig. 1e, 5 mg nanoparticles with number of 5.88e11 were calculated to be able to conjugate with no more than 23 μg of OVA protein ($\overline{V_{max}} = 540$) or 22 μg of BSA protein ($\overline{V_{max}} = 340$)

for the single-layer conjugation. Proteins in different input mass below the saturated value were then added into the nanoparticle solutions respectively, followed by incubation at 4℃ overnight. Cysteine of 0.5 molar ratio to maleimide groups was lastly added into the solution to quench the excess maleimide groups. The mixtures were then purified through ultrafiltration (MWCO = 300 kDa) using DI water as eluent, and finalized to a volume of 200 μL, which were the purified VPNVaxs with different averaged valences.

The final conjugated OVA protein concentrations were quantified by BCA protein assay, and the particle concentrations of the purified VPNVaxs were tested by NanoSight NS300, so with these two data the actual averaged valences could be calculated by Formula IV and furtherly the averaged surface coverage could be calculated by Formula V. The morphology of purified VPNVaxs with different valences were characterized by cryo-EM.

***In vitro* B cell activation.**

To prove the direct activation effect of nanovaccines with surface antigen presentation on B cells, both physically mixing and chemically conjugated nanovaccines (VPNVaxs) with different valences were co-incubated with splenocytes *in vitro* for 7 days, followed by antigen-specific antibody detection on the supernatants. Specifically, C57BL/6 mice were immunized subcutaneously in a prime-boost-regimen with 10 μg OVA formulated with Alum and IMDQ adjuvant. 2 weeks post boost, spleens were harvested, and lymphocytes from one mouse were divided into several groups (2×10^6 cells in total 500 μL medium per group) to be activated by different vaccine formulations (the unified OVA input was 10 μg) in presence of IL-2 (16 ng/ml) at 37℃, 5% CO_2 for 7 days, while half of the supernatants were exchanged with fresh culture medium on day 3. Finally, the supernatants were collected and used for OVA-specific antibody detection assay to evaluate the *in vitro* activation level of B cell.

Histology, immunostaining, and imaging

For lymph node trafficking experiment, OVA protein and BSA protein labelled with Cyanine-5 (Cy5) dye (OVA/Cy5 and BSA/Cy5) were used for the preparation of VPNVax-OVA/Cy5 and VPNVax-BSA/Cy5 with pre-set valences of 50, 100, 200 and 400. After purification and quantification, VPNVaxs were subcutaneous administered via tail base at an equivalent dosage (20 μg per mouse, based on protein). Similarly, for the physical mixing groups, different ratios (equivalent to the ratios of VPNVaxs with different valences) of unmodified OVA/Cy5 proteins and cysteine-blocked PLA nanoparticles were physically mixed and administrated subcutaneously at tail base. At 24h post injection, mice were sacrificed and the inguinal lymph nodes were collected to be photographed by fluorescence imaging. For the trafficking experiment with addition of blank nanoparticles, cysteine-quenched nanoparticles in a series of numbers were added into the VPNVaxs solution before injection. The mean fluorescence intensities of each lymph node were quantified using ImageJ software.

For lymph node retention experiment, OVA/Cy5 was used for the preparation of soluble OVA group, Alum+OVA group (aluminium adjuvant/OVA solution = 1/3, v/v) and VPNVax-200OVA group with the optimal valence of 200. After purification and quantification, VPNVax-200OVA/Cy5 and other groups were subcutaneous administered via tail base at an equivalent dosage (20 μg per mouse, based on protein). Specific group of mice were sacrificed at each desired time point (4h, 2days, 7days and 14days) post injection and the inguinal lymph nodes were collected for histological analysis. Fresh lymph nodes were rapidly merged into optimal cutting temperature compound (OCT compound) in concave tinfoil molds and preserved at -80 ℃. Lymph node samples were eventually sent to outsourcing company for frozen section processing. For immunofluorescent staining, tissue sections were directly stained for cell nucleus using antifade mounting medium with DAPI (Beyotime). The stained tissue sections were scanned using confocal laser scanning microscopy (CLSM) (Carl Zeiss LSM 700). The Cy5(OVA) positive ratios in whole nymph node were quantified using ImageJ software.

For germinal center (GC) of lymph node activation experiment, soluble OVA group, Alum+OVA group (aluminium adjuvant/OVA solution = 1/3, v/v) and VPNVax-200OVA group with the optimal valence of 200 were subcutaneous administered via tail base at an equivalent dosage (20 μg per mouse, based on protein). Specific group of mice were sacrificed at each desired time point (7 days and 14 days) post injection and the inguinal lymph nodes were collected for frozen section processing as above. For immunofluorescent staining, tissue sections were stained for B cell follicles using anti-B220 antibody conjugated with Alexa Fluor 594 (1:100; Biolegend), stained for GC B cells using anti-GL7 antibody conjugated with Alexa Fluor 488 (1:100; Biolegend). The stained tissue sections were scanned using confocal laser scanning microscopy (CLSM) (Carl Zeiss LSM 700). The GL7 positive ratios in whole nymph node were quantified using ImageJ software.

Animal immunization and serum sample collection

The mice were kept in specific pathogen free (SPF) animal lab and operated according

to the guidelines approved by the Animal Care and Use Committee of Jilin University. For the *in vivo* vaccination, 6-week-old C57BL/6 mice were chosen and subcutaneous administered at tail base with different vaccines or control groups. For OVA protein vaccination, due to the inherent intrinsic strong immunogenicity of OVA, the equivalent dosage of different groups was set as 5 μg per mouse based on OVA protein. For BSA protein vaccination, the equivalent dosage of different groups was set as 25 μg per mouse based on BSA protein. For RBD protein vaccination, the equivalent dosage of different groups was set as 10 μg per mouse based on RBD protein. For the groups of direct mixing in OVA/ BSA vaccination, nanoparticles were blended with unmodified proteins at ratio of the optimal valences. For soluble RBD or VPNVax-400RBD formulated with aluminium adjuvant, the aluminium adjuvant (Thermo Fisher Scientific) was added dropwise with constant mixing to the vaccine solution so the final volume ratio of Alum to vaccine is 1:3 (v: v).

After immunization, blood samples (about 200 μL each time without adding any anticoagulant) were collected from the supraorbital vein of mice at specific time points, and then maintained at 37 ℃ for 1h, followed by centrifuging at 900 g for 10 min and collect the supernatant serums, which were sealed and preserved at -20 ℃.

Antigen-specific antibody titer assay

The antigen-specific antibody titer was tested through Enzyme-linked immunosorbent assay. Typically, RBD protein was pre-immobilized on 96-well ELISA plate (Corning) at 10 μg/mL (PBS, 100 μL/well, incubated overnight at 4 ℃). After washed three times with wash buffer containing PBS and 0.05% (v/v) Tween 20, each well was then coated with 200 μL of blocking buffer consisting of PBS and skim milk powder (20 mg/mL) for two hours at room temperature.

Next, mice serums under different dilutions were added into wells to recognize and bind specific antigens, and the maximum dilution with detectable binding activity is set as antibody titer, which can be used to evaluate the antigen-specific antibody strength of the original serum. To be specific, from the initial stock solution, the mouse serum was subjected to a series of 4-fold dilutions, like pipetting 40 μL of existing solution into 120 μL blocking buffer to prepare the next dilution solution. Specially, considering that serums immunized with some vaccines have high specific antibody response, the first dilution applied to the assay would be set relatively higher rather than starting with the initial stock solution. For example, the first dilution could be set as 2^7 (pipetting 1.26 μL of initial serum into 160 μL blocking buffer), 2^10(pipetting 20 μL of 2^7 dilution into 140 μL blocking buffer) or 2^14(pipetting 1.26 μL of 2^7 dilution into 160 μL blocking buffer), followed by a 4-fold dilution series.

For each dilution, a total volume of 100 μL was added to the appropriate wells after wash. Each plate was incubated for 2 hours at room temperature and then washed three times. 100 μL of a 1:5000 dilution of horseradish peroxidase (HRP)–conjugated goat anti-mouse IgG secondary antibody (Invitrogen), goat anti-mouse IgG1 secondary antibody (abcam), or goat anti-mouse IgG2c secondary antibody (abcam)

made in blocking buffer was added to each well for assay of specific IgG, IgG1 or IgG2c antibody in serum. The plate was then incubated with the antibody for one hour at room temperature and then washed five times with wash buffer. Last, 100 μL of tetramethylbenzidene (TMB) (Beyotime) was added to each well for 15 mins at room temperature before stopping the reaction with 100 μL of 2N sulfuric acid. The resulting plate readouts were measured at a wavelength of 450 nm.

For statistical analysis of antibody titer, in general, OD value of each well lower than 0.03 (could be adjusted referring to the blank control absorption value) would be judged as undetectable dilution, and the dilution before which is set as antibody titer value.

Authentic SARS-CoV-2 neutralization assays

An authentic SARS-CoV-2 neutralization assay was performed using a cytopathic effect (CPE) assay in a biosafety level 3 laboratory. The titers of neutralizing antibodies in serum of mice immunized with different groups of vaccines were determined by micro neutralization test. In the experiment of mice, the neutralizing antibody titers of serum were measured on day 40/80 after the prime-boost immunization on day 21. To evaluate the neutralization effect of SARS-CoV-2 infection, 50μL of 100×$TCID_{50}$ Delta (CSTR.16698.06.NPRC6.CCPM-B-V-049-2105-6) or Omicron BA.1 (SARS-CoV-2 strain Omicron CoV/human/CHN_CVRI-01/2022) strain solution was pre-incubated with equal volume of 20× diluted serum. After incubation at 37 °C for 1 hours, 100μL Vero E6 cell suspension (5×10^3) was added to the above mixture. On the 5th day after infection, cytopathic effect (CPE) was recorded under microscope, and neutralizing antibody titer was calculated by Reed-Muench method.

Peripheral blood analysis of T cell responses

C57BL/6 mice were immunized on day0 and peripheral blood samples were collected on day 7. Red blood cells were lysed using ACK Lysing Buffer. Then samples were

incubated with 2ug/ml RBD peptide pool in presence of cell activation cocktail (with Brefeldin A) for 5 h at 37 ℃. Samples were stained with anti-mouse CD3, CD4 and CD8 antibodies, and treated with intracellular staining permeabilization wash buffer. Permeabilized samples were then stained for IFN-γ and analyzed by standard flow cytometry protocols.

BMDC activation *in vitro*

For BMDCs activation assay, BMDCs were seeded in 24-well plates at the density of 3×10^5 cells per well. 60 μg of assembled nanoparticles (30 mg/mL) made of PDLLA, PLLA and PDLA were added into each well. After 6 h incubation, cells were gently harvested and stained with anti-mouse PE-CD11c, anti-mouse APC/Cy7-MHC-II, anti-mouse APC-CD80 and anti-mouse PE/Cy7-CD86 for flow cytometry analysis. All the staining procedures were performed on ice to avoid unnecessary stimulation to BMDCs. Flow cytometry tests were performed by a flow cytometer (BD FACSCelesta).

Statistical analysis

All experiments were performed at least three times and expressed as means ± standard deviation (SD). Student's t-test was used to analyze statistical significance between two groups. ns means no significance. *$p < 0.05$ was considered statistically significant, whereas **$p < 0.01$, ***$p < 0.001$ and ****$p < 0.0001$ were considered highly and extremely significant.

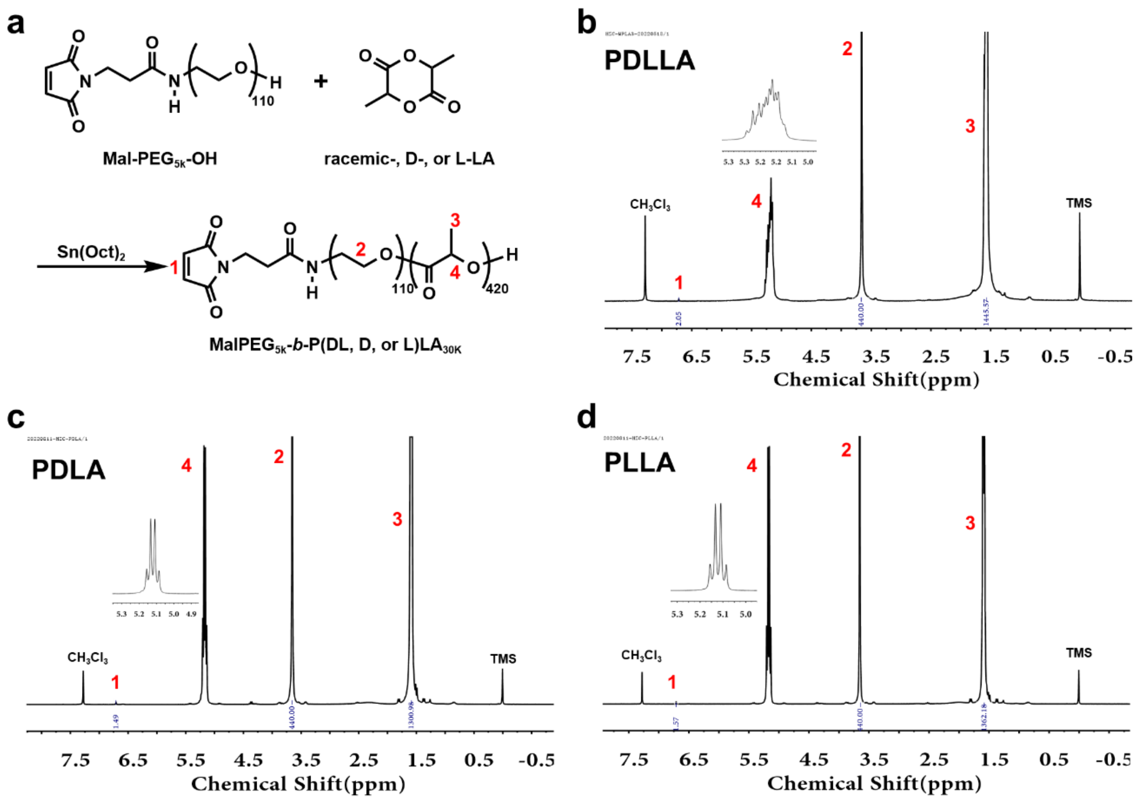

Figure S1. Synthesis and characterization of maleimide-terminated polyethylene glycol-block-poly (racemic-, D-, or L-lactide acid) (MalPEG-b-P (DL, D, or L) LA).

(a) Synthetic route of MalPEG-b-P (DL, D, or L) LA.

(b-d) ^{1}H NMR spectrum of b) PDLLA, c) PDLA and d) PLLA with chloroform-d as the solvent. The peak at about 3.6 ppm from PEG_{5k} was set as integral value of 440. The peak at about 6.7 ppm represented the preservation of maleimide groups.

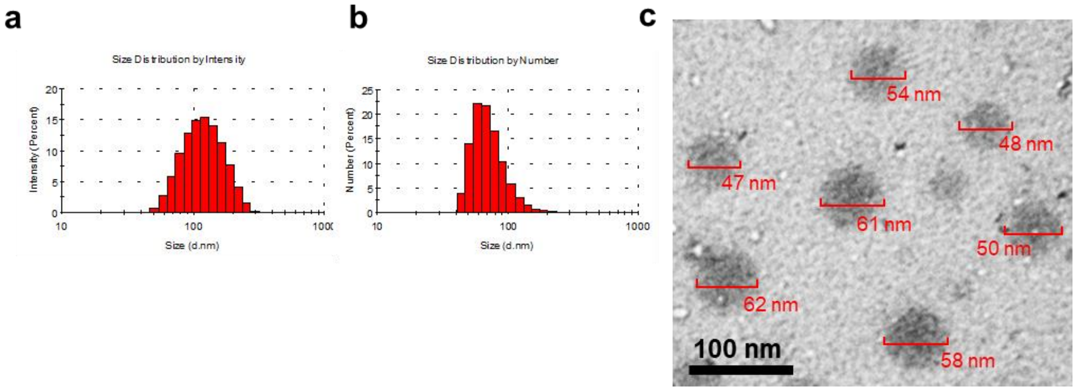

Figure S2. Size and morphology of MalPEG-*b*-PDLLA nanoparticles.

(a-b) a) Intensity-distribution size histogram and corresponding b) number-distribution size histogram of MalPEG-b-PDLLA nanoparticles.

(c) Transmission electron microscopy (TEM) images of MalPEG-b-PDLLA nanoparticles. Scale bar = 100 nm.

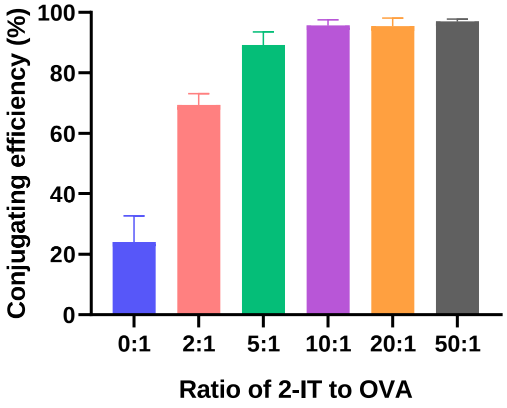

Figure S3. Conjugating efficiency of OVA onto PLA vectors when modified by different ratio of 2-IT.

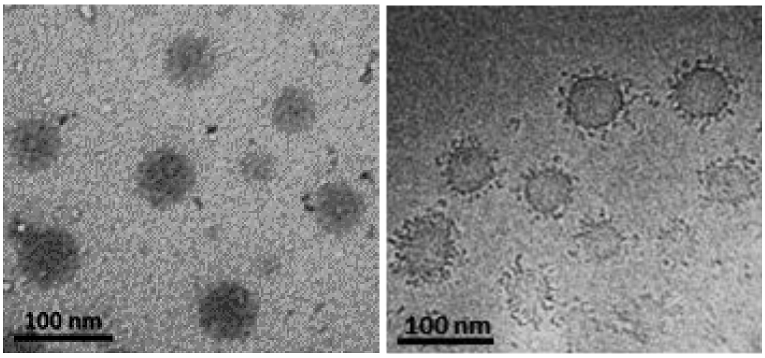

Figure S4. Morphological comparison of PLA-NP before and after conjugating with OVA proteins.

Left panel: pure PLA-NP. Right panel: 2mg PLA-NP conjugating with 10μg OVA proteins. Scale bar = 100 nm.

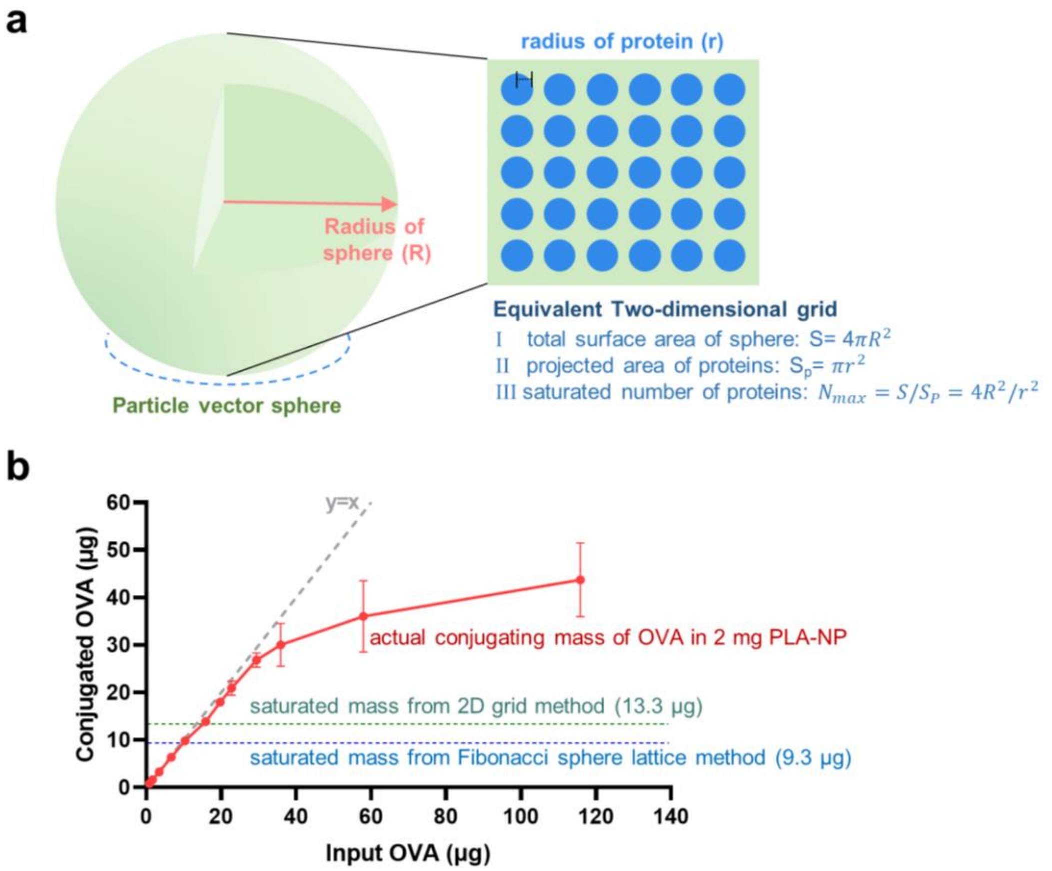

Figure S5. Two-dimensional grid method to calculate the saturated number of proteins for conjugation.

(a) the outer surface of spherical vector is equivalent to a two-dimensional plane, and the saturated conjugating number of the protein is calculated by the ratio of S and S_p.

(b) relation curve of the input mass and the actual conjugating mass of OVA (co-incubated with 2 mg NP).

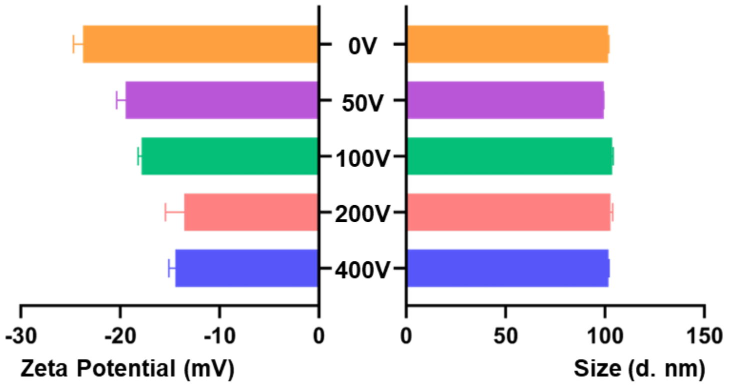

Figure S6. Sizes and zeta potentials of VPNVaxs-OVA with different valences. Valences of VPNVaxs-OVA were set as 0 (0V), 50 (50V), 100 (100V), 200 (200V) and 400 (400V). Results were detected by Zetasizer Nano ZS (Malvern).

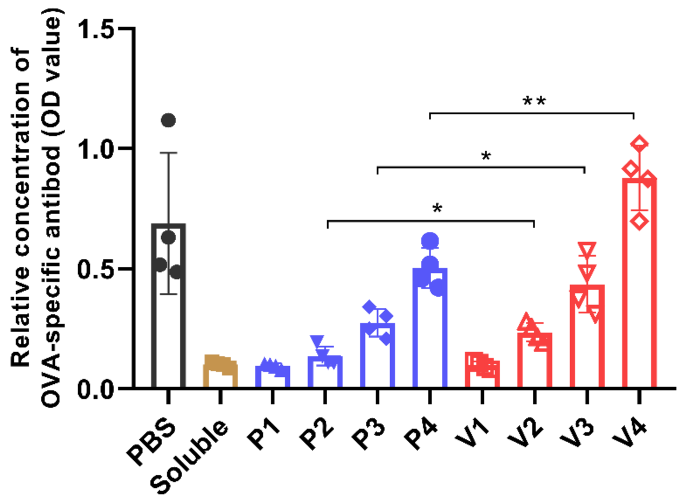

Figure S7. *In vitro* B cells restimulation by antigen in different formulations. Results of the relative concentrations of OVA-specific antibody in supernatants from different groups. Valences of physical mixing nanovaccines and VPNVaxs were set at 50 (P1 and V1), 100 (P2 and V2), 200 (P3 and V4) and 400 (P4 and V4). The relative concentrations were expressed by OD values obtained from the ELISA test. Data were performed as mean ± SD (n = 4) and were analyzed by student's t-test. *p < 0.05; **p < 0.01.

Supplementary explanation: As showed in Figure 2a, immunized splenocytes were co-incubated with antigens *in vitro*, where antigen-specific memory B cells were stimulated and would differentiate into effector B cells to produce antigen-specific antibodies. However, two interference factors need to be taken into account when comparing the final antibody amount. Firstly, since the immunized splenocytes had been not further sorted, the cells involved in co-incubation included the existing antigen-specific effector B cells in addition to the memory B cells, which explained why appreciable concentration of antibodies could be detected from the PBS group. Secondly, the addition of antigens, while working as stimulating agents, would inevitably bind to antigen-specific antibodies in the co-incubation system, and those antigen-bound antibodies would be undetectable in the ELISA, which explained why the relative concentrations of antibody detected from the soluble antigen group were significantly lower than those in the PBS group. Therefore, in the antigen-added groups, antibody concentrations could be detected only if the antigen formulation was highly effective in activating B cells to produce sufficient amounts of antibodies to override the interference of the added antigens. The antibody concentrations among soluble antigen group, physical adsorbed nanovaccines groups and VPNVaxs groups were comparable, because the amount of the added antigens of these groups were consistent, and results showed that nanovaccines with high-valence antigen displaying on the surface had advantage in activating B cells.

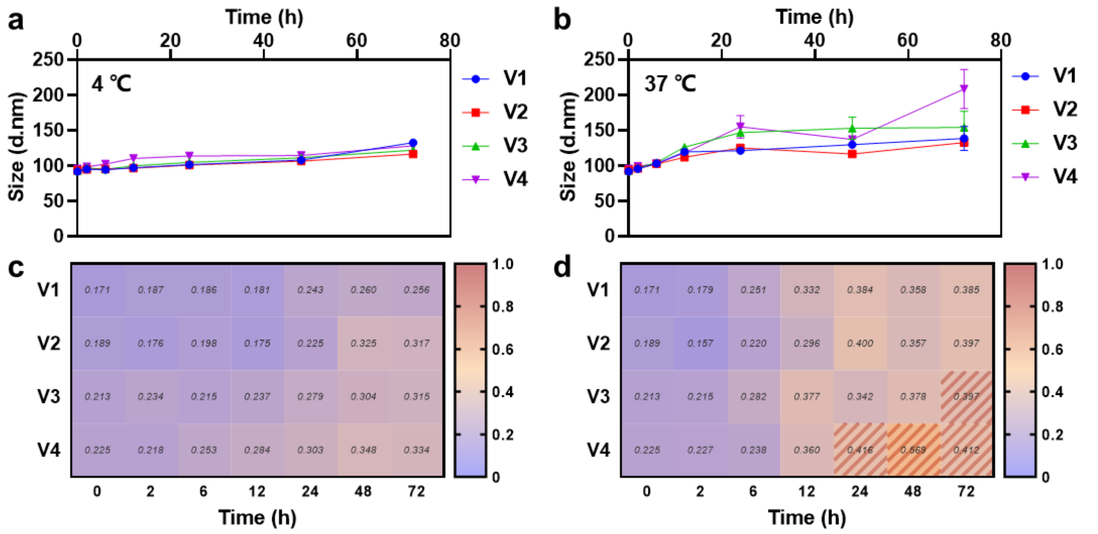

Figure S8. The size stability of VPNVaxs-OVA with different valences.

Valences of VPNVaxs-OVA were set as 50 (V1), 100 (V2), 200 (V3) and 400 (V4), and incubated in PBS (0.01M, 7.4) in presence of FBS (5%) at 4 ℃ or 37 ℃ for 72h.

(a, b) Z-averaged sizes of VPNVaxs-OVA from 0 h to 72 h at a) 4 ℃ or b) 37 ℃ presented as the mean ± SD (n = 3).

(c, d) PDI of size distributions of VPNVaxs-OVA from 0 h to 72 h at c) 4 ℃ or d) 37 ℃, whose mean values (n = 3) were presented in the heat map. The red dashed line in some cells indicated that the sample became polydisperse and its data quality was too poor for distribution analysis.

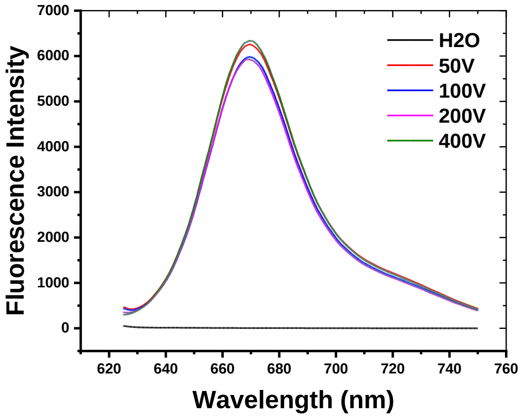

Figure S9. Fluorescence emission spectrums of VPNVaxs-OVA/Cy5 with different valences.

Valences of VPNVaxs-OVA/Cy5 were set as 50 (50V), 100 (100V), 200 (200V) and 400 (400V). For fluorescence intensity determination, VPNVaxs-OVA/Cy5 with valences of 50, 100, 200 and 400 were prepared following the method introduced above, and their fluorescence emission spectrums were detected by fluorescent spectrometer set with excitation wavelength of 600 nm and emission wavelength from 625 nm to 750 nm.

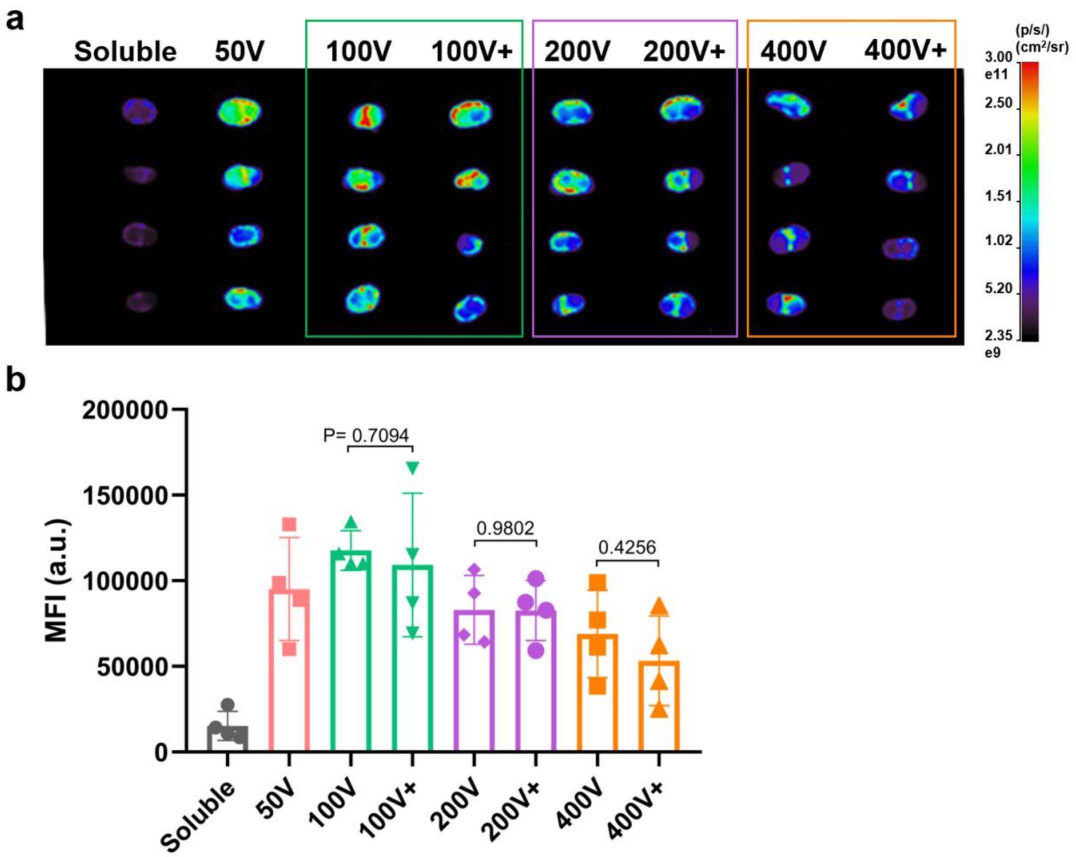

Figure S10. Trafficking of VPNVaxs with addition of blank nanoparticles in mouse lymph node.

(a) *Ex vivo* fluorescence imaging of inguinal lymph nodes at 24h post s.c. administered of VPNVax-BSA/Cy5 with addition of blank nanoparticles as Table S3. 50V, 100V, 200V and 400V represent VPNVax-BSA/Cy5 with valences of 50, 100, 200 and 400, and 100V+, 200V+ and 400V+ represent VPNVax-BSA/Cy5 with different valences of 100, 200 and 400 with addition of blank nanoparticles as Table S3. Scale bar ranged from 2.35e9 to 3.00e11 p/s/cm^2/sr.

(b) Quantification of mean fluorescence intensity of a). Data were performed as mean ± SD (n = 4) and were analyzed by student's t-test.

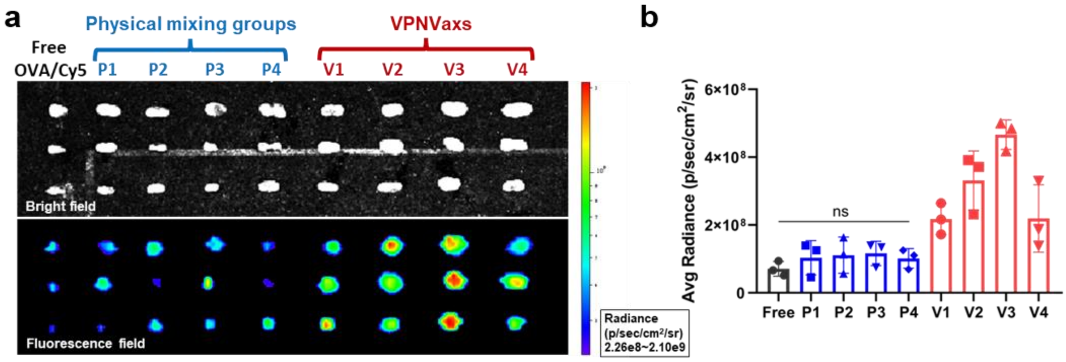

Figure S11. Trafficking of OVA/Cy5 in different formulations to mouse lymph nodes.

Valences of physical mixing groups and VPNVaxs were set at 50 (P1 and V1), 100 (P2 and V2), 200 (P3 and V4) and 400 (P4 and V4).

(a) *Ex vivo* bright field and fluorescence imaging of draining lymph nodes at 24h post s.c. administered of OVA/Cy5 in different formulations. Scale bar ranged from 2.26e8 to 2.10e9 p/sec/cm^2/sr;

(b) Quantification of mean fluorescence intensity of a). Data were performed as mean ± SD (n = 3) and were analyzed by student's t-test.

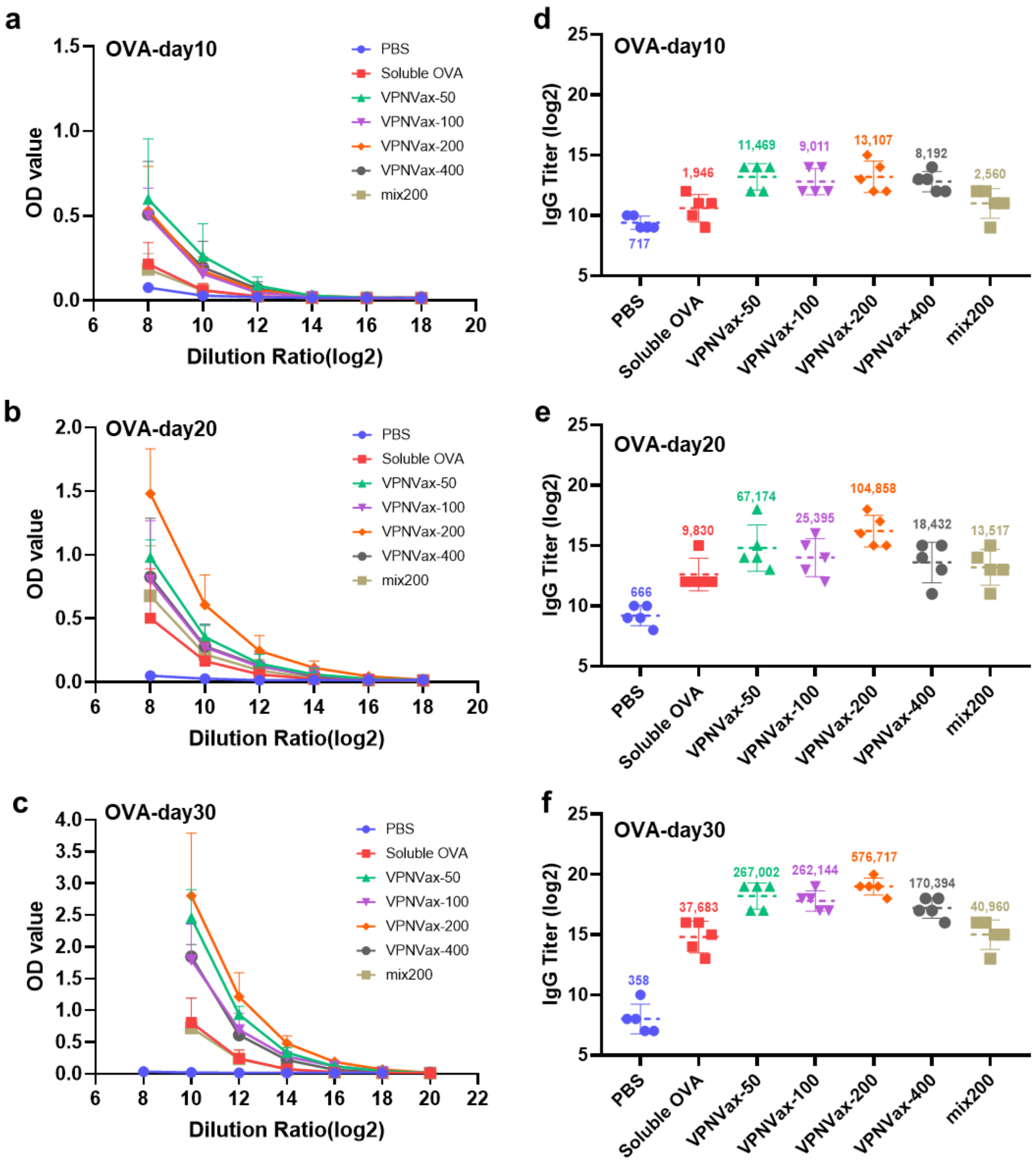

Figure S12. OVA-Specific IgG antibody titers induced by VPNVaxs-OVA with different valences.

(a-c) The changing curves of absorption intensity (OD value, optical density) vs dilution ratio (log2) of serums in OVA-specific antibody titer assay for serum samples on a) day 10, b) day 20, and c) day 30 after the day 0 immunization.

(d-f) OVA-specific IgG antibody endpoint titers on day d) 10, e) day 20 and f) day 30. All data above presented as the mean ±SD (n = 5).

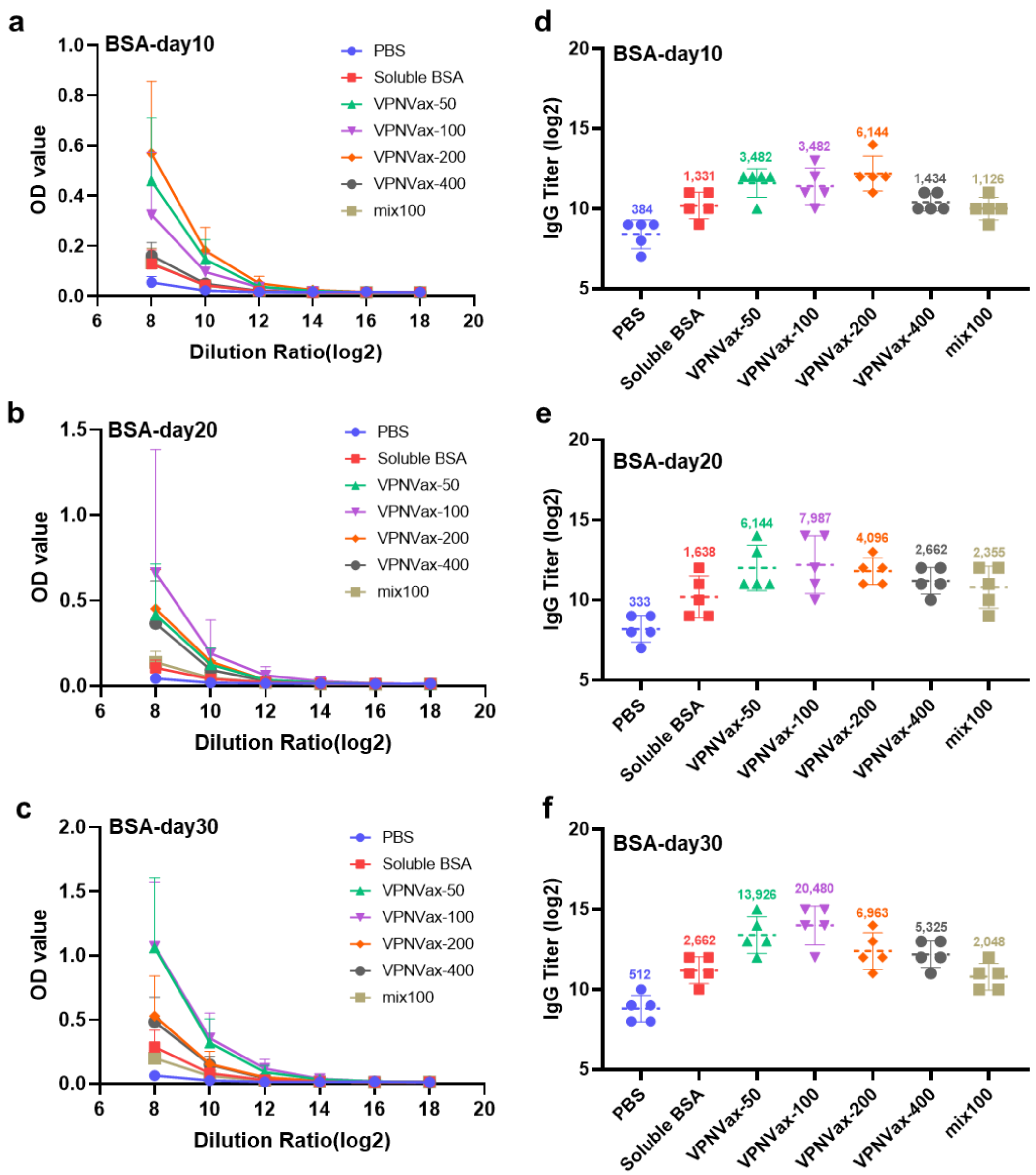

Figure S13. BSA-Specific IgG antibody titers induced by VPNVaxs-BSA with different valences.

(a-c) The changing curves of absorption intensity (OD value, optical density) vs dilution ratio (log2) of serums in BSA-specific antibody titer assay for serum samples on a) day 10, b) day 20, and c) day 30 after the day 0 immunization.

(d-f) BSA-specific IgG antibody endpoint titers on d) day 10, e) day 20 and f) day 30. All data above presented as the mean ± SD (n = 5).

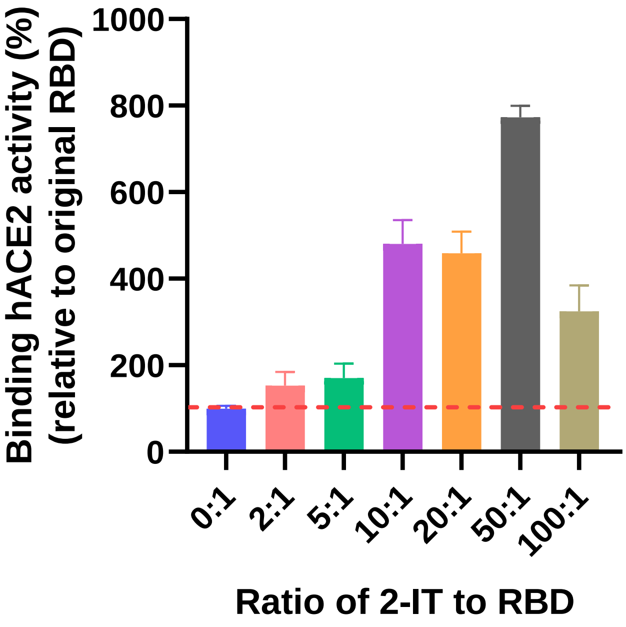

Figure S14. Assay of the binding activity of modified RBD to human ACE2. The averaged absorption value from group of naïve RBD protein (0:1) was set as 100% activity standard. Interestingly, higher modifying ratios (from 10:1 to 100:1) would significantly enhance the binding activity (from 400% to 800%), which could be attributed to the crosslinking of overmodified RBD proteins that enhanced binding activity through clustering effect. All data are presented as the mean ± SD (n = 3);

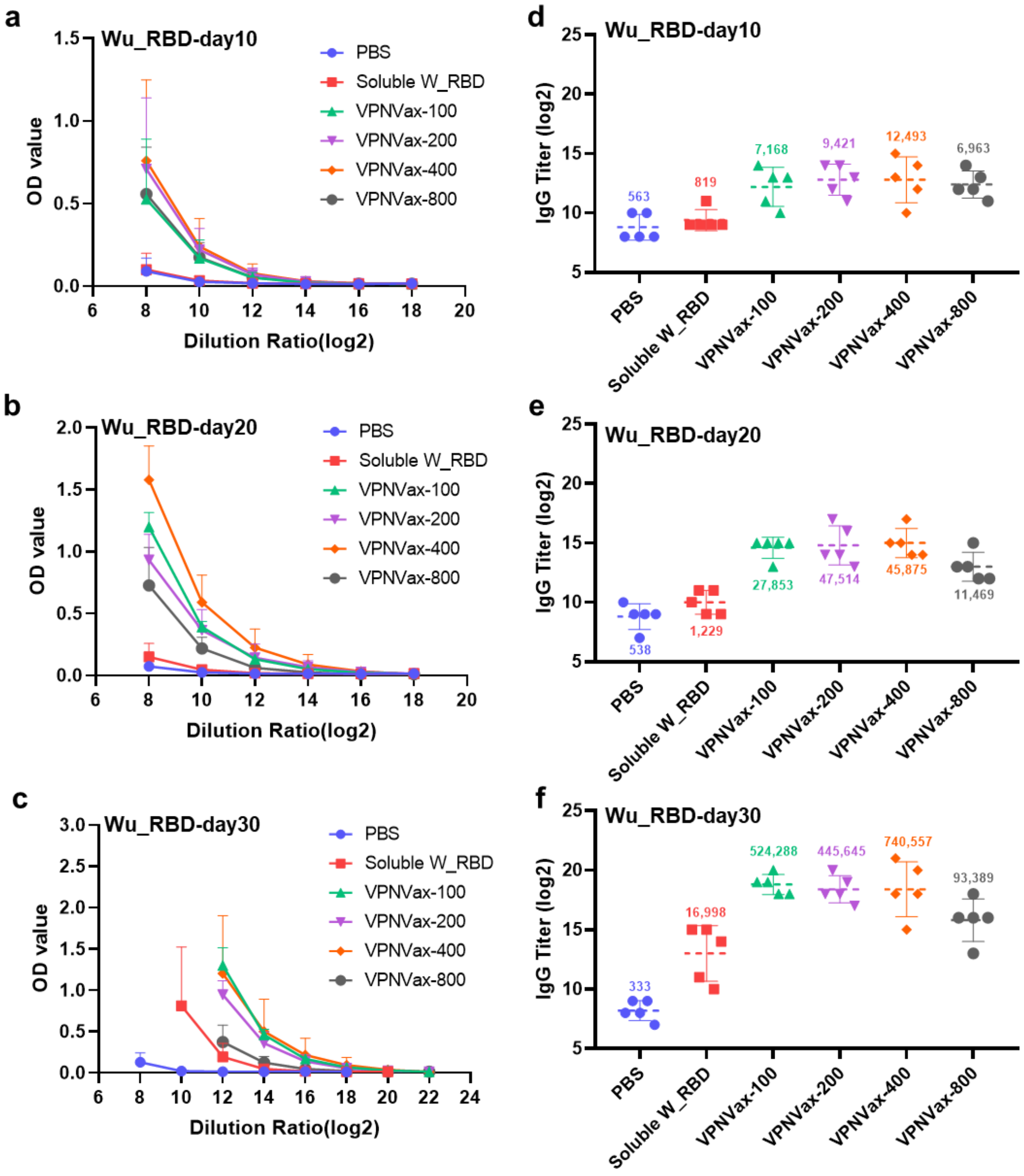

Figure S15. Wu_RBD-Specific IgG antibody titers induced by VPNVax-WRBD with different valences.

(a-c) The changing curves of absorption intensity (OD value, optical density) vs dilution ratio (log2) of serums in Wu_RBD-specific antibody titer assay for serum samples on a) day 10, b) day 20, and c) day 30 after the day 0 immunization.

(d-f) Wu_RBD-specific IgG antibody endpoint titers on d) day 10, e) day 20 and f) day 30. All data above presented as the mean ± SD (n = 5).

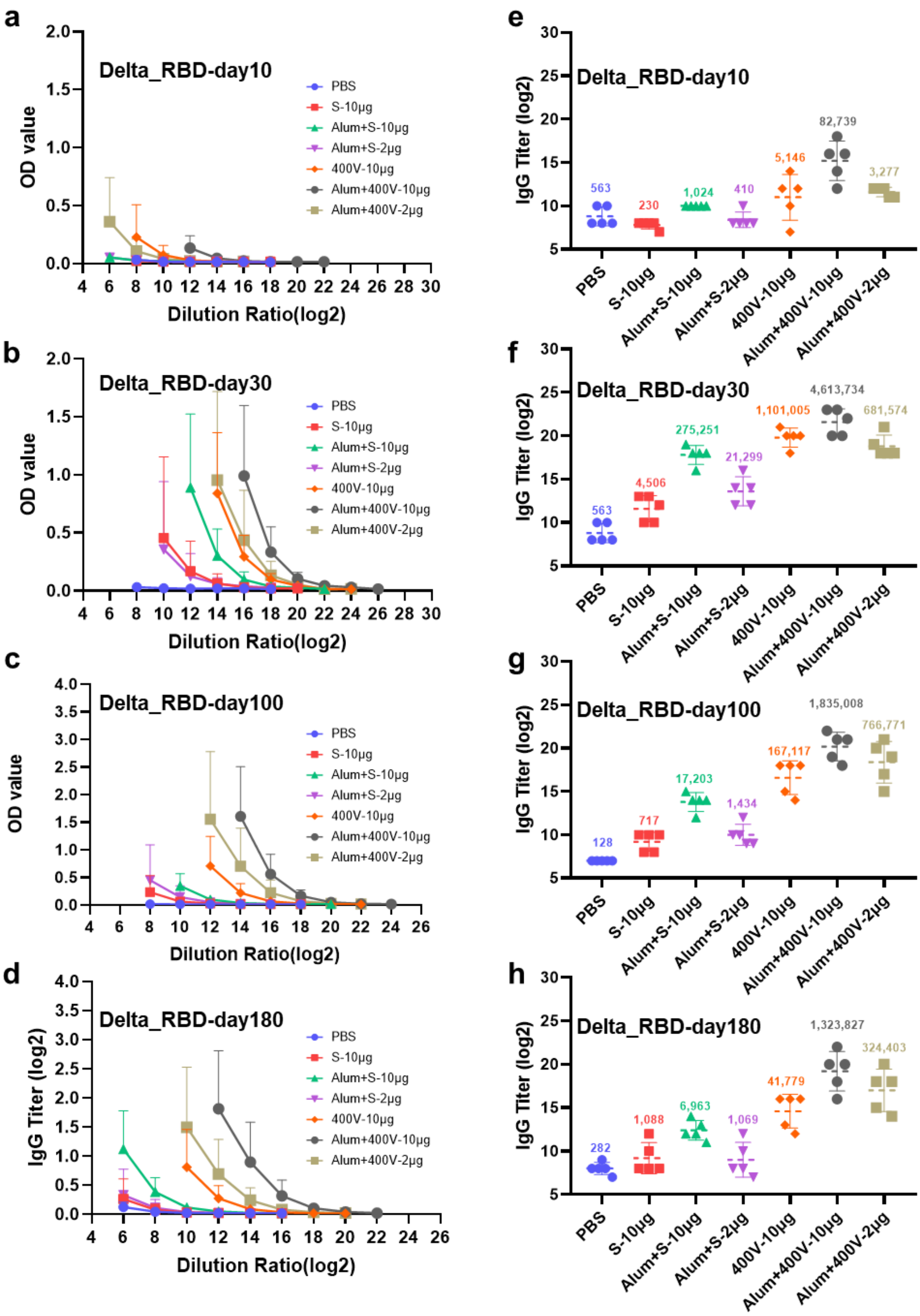

Figure S16. Delta_RBD-Specific IgG antibody titers induced by VPNVax-DRBD formulated with Alum-adjuvant.

(a-d) The changing curves of absorption intensity (OD value, optical density) vs dilution ratio (log2) of serums in Delta_RBD-specific antibody titer assay for serum samples on a) day 10, b) day 30, c) day 100 and d) day 180 after the day 0 immunization.

(e-h) Delta_RBD-specific IgG antibody endpoint titers on e) day 10, f) day 30, g) day 100 and h) day 180. All data above presented as the mean ± SD (n = 5).

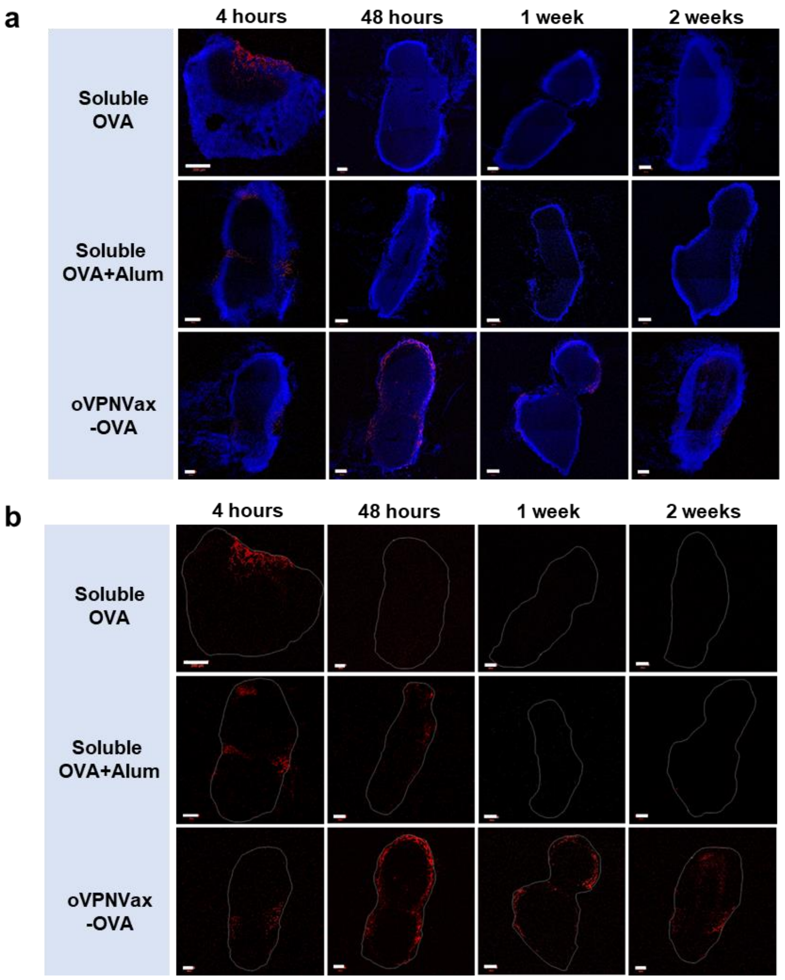

Figure S17. CLSM images of complete lymph nodes sections in immunofluorescence analysis of OVA/Cy5 distribution and retention.

(a) Merged-channel CLSM images of OVA/Cy5 distribution in lymph nodes of different treatment groups at different time points (Scale bar = 200 μm). OVA/Cy5 channel is set as red while DAPI channel is set as blue in the images.

(b) Single-channel CLSM images of OVA/Cy5 distribution in lymph nodes of different treatment groups at different time points (Scale bar = 200 μm). OVA/Cy5 channel is set as red and drawn white lines indicate the outer contour of lymph nodes.

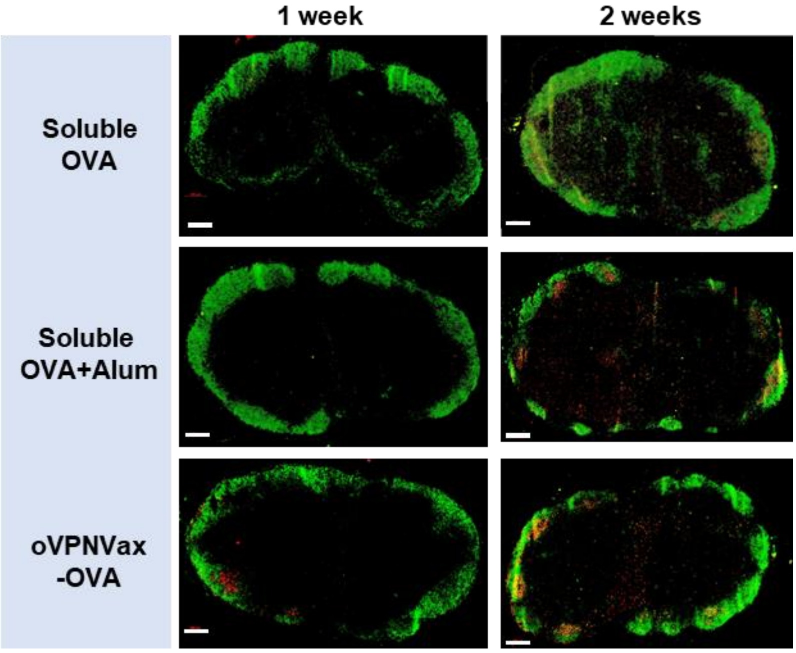

Figure S18. CLSM images of complete lymph nodes sections in immunofluorescence analysis of GCs activation.

CLSM images of GCs activation in lymph nodes of different treatment groups at different time points (Scale bar = 200 μm). GC B cells ($GL7^+$) channel is set as red while B cell follicles ($B220^+$) channel is set as green in the images.

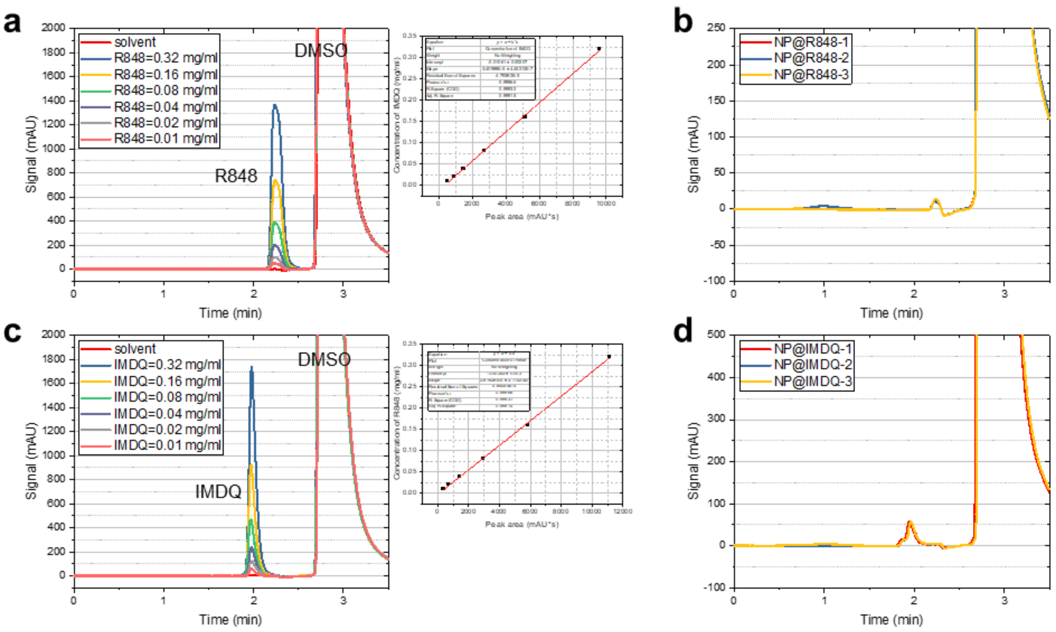

Figure S19. HPLC tests on drug loading efficiencies of oVPNVax@r848 and oVPNVax@IMDQ.

Tested samples were dissolved in acetonitrile and DMSO (5%), and the mobile phase of HPLC was a mixture of water (20%) and acetonitrile (80%). Retention times of R848 and IMDQ were 2.23 min and 1.97 min respectively. Test wavelength: 214 nm.

(a, c) HPLC chromatograms and standard curves (right panel, R>0.999) of standard samples of a) R848 and c) IMDQ in different concentrations.

(b, d) HPLC chromatograms of b) oVPNVax@R848 and c) oVPNVax@IMDQ. The averaged peak areas of oVPNVax@R848 and oVPNVax@IMDQ samples were 95.8 mAU*s and 373.7 mAU*s respectively, which were ultimately converted into averaged drug loading efficiencies of 0.14% and 0.64% respectively (n = 3).

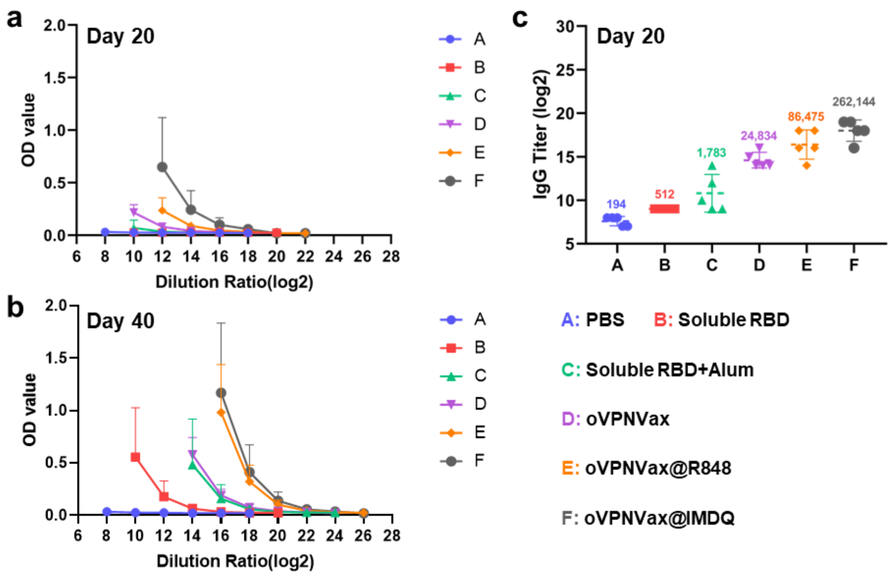

Figure S20. Delta_RBD-Specific IgG antibody titers induced by oVPNVax-DRBD loading with R848 or IMDQ.

(a-b) The changing curves of absorption intensity (OD value, optical density) vs dilution ratio (log2) of serums in Delta_RBD-specific antibody titer assay for serum samples on a) day 20 and b) day 40 after the day 0 immunization and day 21 boost. **(c)** Delta_RBD-specific IgG antibody endpoint titers on day 20. Endpoint titers derived from antigen-specific antibody titer assays are plotted, with y-axis representing the log base 2 of titers and average titer values labeled below the plots. All data above presented as the mean ± SD (n = 5).

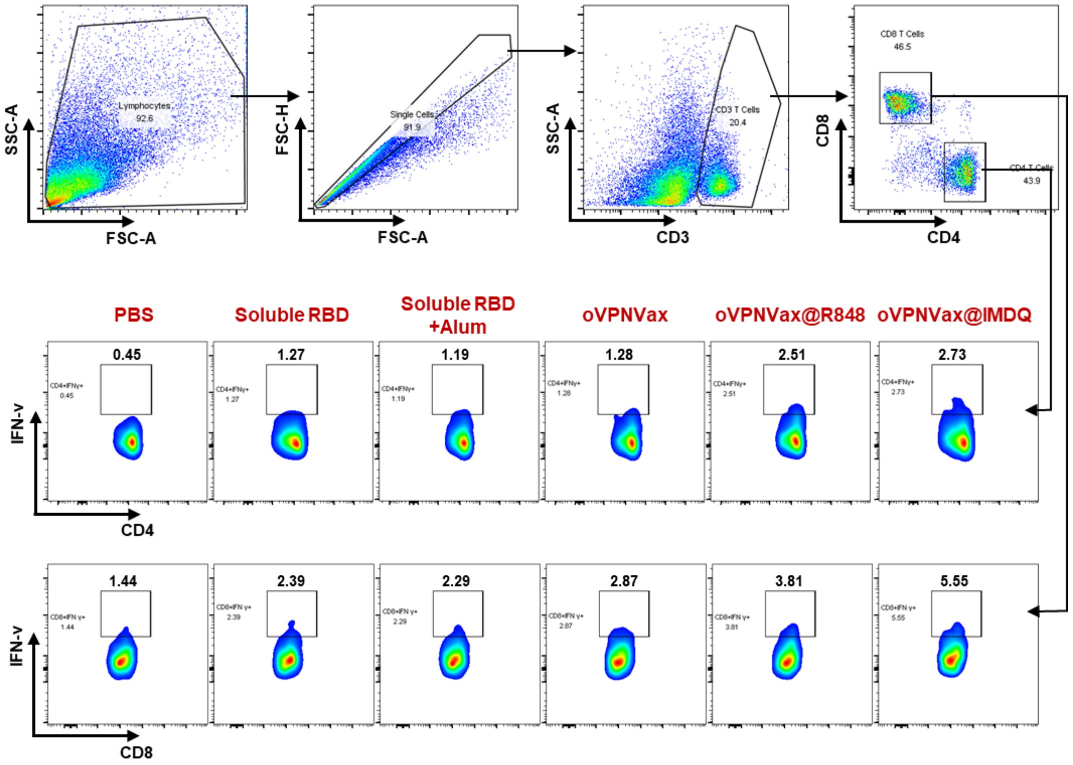

Figure S21. Flow cytometry gating strategy of IFN-γ$^+$CD4$^+$ and IFN-γ$^+$CD8$^+$ T cells in blood after immunized by different vaccines.

C57BL/6 mice were immunized on day0 for one shot and blood samples were collected on day 7 for flow cytometry analysis.

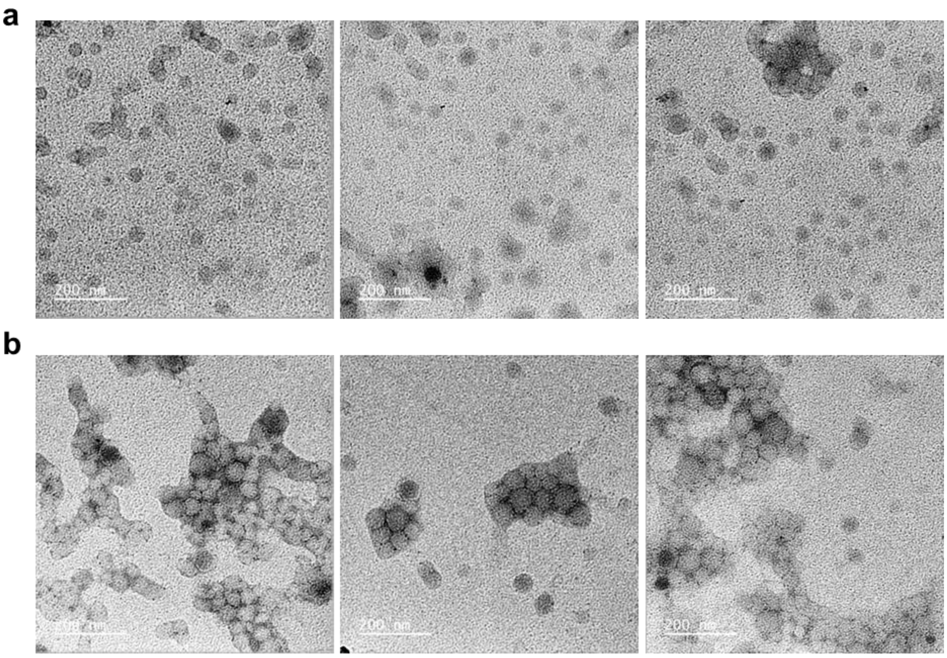

Figure S22. TEM morphological characterization of nanoparticles assembled by MalPEG-*b*-PLLA and MalPEG-*b*-PDLA.

(a-b) Three TEM images of parallel conditions were presented to show the morphological characteristics of a) MalPEG-b-PLLA and b) MalPEG-b-PDLA nanoparticles. As crystalline polymers with strong intermolecular interaction, these two types of nanoparticles, while remaining spherical, tended to aggregate, especially for MalPEG-b-PDLA nanoparticles. Scale bar = 200 nm.

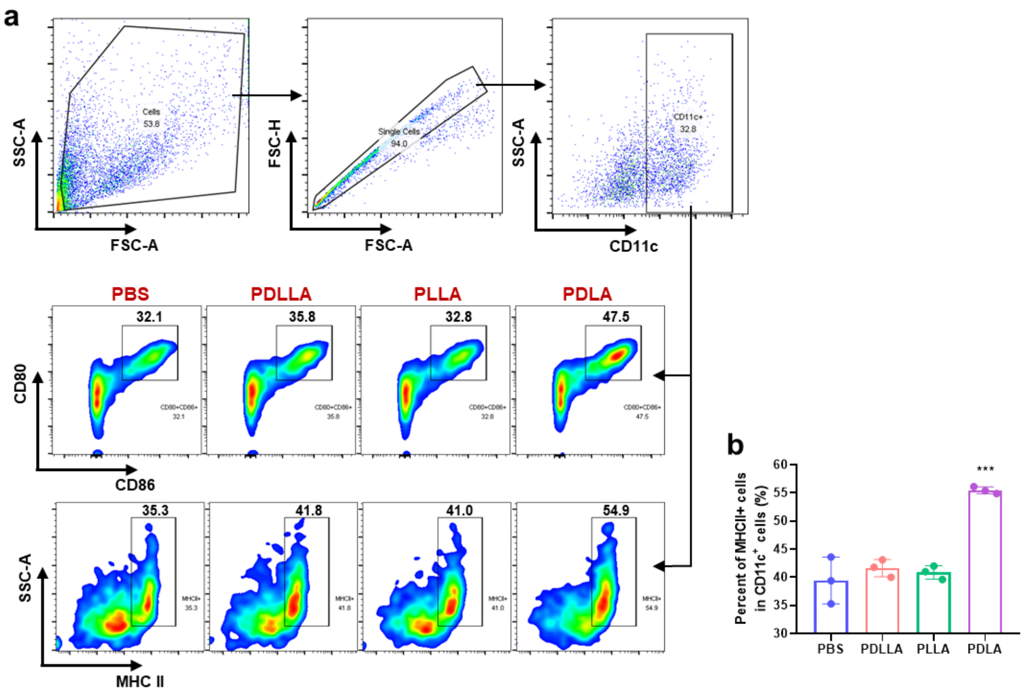

Figure S23. Flow cytometry gating strategy of CD80$^+$CD86$^+$ and MHC II$^+$ cells in CD11c$^+$ cells of BMDC after co-incubated with different materials *in vitro*.

(a) Flow cytometry gating strategy.

(b) Statistic of activated populations (MHC II$^+$) in BMDC treated with different materials. All data are presented as the mean ± SD (n = 3). *p < 0.05; **p < 0.01; ***p < 0.001.

Table S1. Percent of particles in different size bins (number distribution).

Size (D*. nm)	percent (p. %)
43.8	3.9
50.7	14
58.8	22.1
68.1	21.8
78.8	16.4
91.3	10.3
106	5.79
122	2.98
142	1.44
164	0.679
190	0.308
220	0.12
255	0.0334
295	0.0054
342	0.000184

* D (diameter)=2R (radius)

** $\sum p_i \cdot R_i^2$ =1462.4 (nm^2)

Table S2. The average saturated valences of different proteins calculated from two methods.

	OVA (d≈ 5.5 nm)	BSA (d≈ 7.0 nm)	RBD (d≈ 4.1 nm)
**2D grid method**	774	477	1392
**Fibonacci sphere lattice method**	543	336	978

Table S3. Particle numbers of VPNVaxs-BSA with different valences.

Mass of BSA proteins per dose	25 μg (Mw= 66 kDa)			
Averaged valence of VPNVax	**50**	**100**	**200**	**400**
Number of particles per dose ($\times 10^{12}$)	4.56	2.28	1.14	0.57
Number of supplementary blank particles ($\times 10^{12}$)	-	2.28	3.42	3.99
